# Balancing landscape values and tourism choices: Integrating participatory mapping and the IPBES Values Typology

**DOI:** 10.1007/s13280-024-02112-6

**Published:** 2025-01-03

**Authors:** Liliana Solé, Kyle P. Hearn, Tahjudil Witra, Alex M. Lechner, Nora Fagerholm

**Affiliations:** 1 Department of Geography and Geology, University of Turku, 20014 Turku, Finland; 2 Department of Geography, Universitat Autònoma de Barcelona, Barcelona, Spain; 3 Department of Human Sciences and Education, Universidad Pública de Navarra, Pamplona, Spain; 4 Monash University Indonesia, Green Office Park 9, The Breeze BSD City, Kabupaten Tangerang, Banten 15345 Indonesia

**Keywords:** Biosphere reserve, Intrinsic, Instrumental, Landscape values, Relational, Tourism development preferences

## Abstract

The United Nations Educational, Scientific and Cultural Organization (UNESCO) Biosphere Reserves aim to balance nature and human coexistence, but increasing tourism challenges landscape management. The Intergovernmental Science-Policy Platform on Biodiversity and Ecosystem Services (IPBES) stresses the importance of understanding stakeholder values for effective management of natural spaces through a typology of intrinsic, instrumental, and relational values. This study applies this IPBES typology to tourism preferences in the Archipelago Sea Biosphere Reserve (ABR) using Public Participation Geographic Information Systems (PPGIS). A 2022 survey gathered data from 690 locals, recreationists, and tourists revealing intrinsic values as the most significant. Locals prioritized intrinsic and relational values, while recreationists and tourists emphasized relational and instrumental values. The study highlights a preference for small-scale tourism that preserves landscapes and heritage, illustrating the complexity of aligning stakeholder values in tourism and conservation. Insights from this research guide policymaking to better accommodate diverse values in protected areas.

**Supplementary Information:**

The online version contains supplementary material available at 10.1007/s13280-024-02112-6.

## Introduction

Landscapes are the result of human-nature interactions and comprise both physical and human-made features. Key to understanding the anthropocentric relationship with landscape, are not only the human-made features found on the physical landscape, but also the perceptions and understanding that people have of a landscape (Council of Europe 2000). These perceptions vary based on the cultural background and personal experiences in the landscape (Brown and Raymond 2007; Scott et al. 2009). Stakeholder groups and communities, even individuals, attribute diverse values to landscapes, such as recreational, aesthetic, ecological, cultural, and economic values (Jones et al. 2016; Ernoul et al. 2018). For policy makers, advancing balanced human-nature relationships, recognizing, and understanding diverse landscape values are crucial for promoting sustainable development that balances the environmental and social needs and economic activity (Selman 2006).

Several international institutions and agreements have emphasized the importance of understanding stakeholder values of landscape. The European Landscape Convention (ELC) of 2000 espouses policy and research that contribute to the identification and assessment of landscape values. Also, the United Nations Educational, Scientific and Cultural Organization (UNESCO) in its management framework of world cultural landscapes, emphasizes that successful landscape management should recognize ‘cultural and ecological values’, ‘be economically beneficial’, and ‘contribute to a sustainable society’ (UNESCO 2017). The Intergovernmental Science-Policy Platform on Biodiversity and Ecosystem Services (IPBES) is an organization whose purpose is to “provide scientific assessments about the state of knowledge regarding the planet’s biodiversity, ecosystems and the contributions they make to people, as well as options and actions to protect and sustainably use these vital natural assets” (IPBES 2019). The recently published IPBES Values Assessment (2022a) underscored the low uptake of socio-cultural valuation in decisions concerning land and resources (less than 5%).

The Values Assessment by IPBES presents a typology that combines worldviews and knowledge systems with various values and indicators for valuing nature's benefits to people (IPBES 2022a). This framework outlines a categorization of three groups of the Specific Values Typology (from now on called IPBES Values Typology), to reflect nature’s importance to people: intrinsic, instrumental, and relational. Instrumental values relate to ecosystem services. They satisfy an anthropocentric view and refer to the benefits that natural entities provide to human well-being, such as food, clean water, or recreational values. Intrinsic values refer to the inherent value of nature and natural entities, regardless of their usefulness or value to humans such as species or habitats that are innately worthy of protection. Relational values refer to the connections and non-replaceable relationships that people (even through different generations) have with nature including cultural, spiritual, and aesthetic values, as well as the sense of place, identity, and community that these connections provide (IPBES 2022a).

In the context of protected areas, the coordination of diverse values among stakeholder groups, particularly the clash between economic development, tourism, and environmental conservation, often leads to contradictions. Today a more nuanced, multidisciplinary, dynamic approach to managing protected areas has emerged that integrates both 'nature and people', whereby an emphasis is placed on a shared, sustainable, and resilient human-nature relationship (Mace 2014). This ‘nature and people’ conservation approach is key to understanding and identifying the diversity of values related to natural resources, land use and sustainability. However, the different values can often be challenging to identify due to their subjective nature (Jacobs et al. 2016; Pascual et al. 2017; IPBES 2022b). Addressing this plurality of perspectives, the IPBES framework of nature’s Values Typology emphasizes recognizing the richness of the relationships between people and nature across different stakeholders. Several areas in this framework, however, in our view require further investigation. Firstly, there is a need to operationalize this IPBES Values Typology in empirical research to transform land and resource planning into a more sustainable direction. The second need relates to the application of value pluralism in the context of protected areas such as Biosphere Reserves and their management. Last, there is a need to understand how different stakeholder groups value these landscapes, as to identify possible conflicts or synergies.

UNESCO Biosphere Reserves (UNESCO 2017) constitute a network of protected areas designed to integrate biodiversity and biocultural diversity conservation, demonstrate sustainable development, and promote education, research, and monitoring (Winkler 2019). These reserves consist of a core conservation zone, a buffer zone, and a broader transition zone that often includes various human activities, including economic ones like tourism. The increasing tourism in these areas raises sustainability concerns due to potential conflicts between the values of tourists and residents (Kala and Maikhuri 2011; Hoppstadius and Dahlström 2015).

While there are other methods to study landscape values such as the integrated Cultural Values Model approach (Stephenson 2008), stakeholder mapping (Villegas-Palacio et al. 2016), or quantitative landscape preference-value correlation (Zhang et al. 2021), Public Participation Geographic Information Systems (PPGIS) (Fagerholm et al. 2022) is unique in that it aggregates qualitative information acquired from individuals regarding place descriptions into a context of mapped, geospatial data. These data can be merged to analyse and identify patterns across many stakeholder groups. In doing so, it has great potential to advance more sustainable land and resource management (Brown and Kyttä 2014; Plieninger et al. 2018; Fagerholm et al. 2021). Furthermore, it is important to acknowledge the diversity and plurality in values between different community segments. Variations in social values appear across different groups within the population, necessitating an understanding and appreciation of these differences both within and among these segments (Lechner et al. 2020; Lourdes et al. 2024). Conflicts may arise when individuals or stakeholder groups, having different interests and development preferences, assign different social values to the same location (Hausner et al. 2015; Lechner et al. 2020, Lourdes et al. 2024).

In this study, we apply a PPGIS survey approach in the context of the Archipelago Sea Biosphere Reserve (ABR), located in Southwestern Finland. The landscape of the ABR is particularly sensitive to changes. This is evident from numerous endangered species, shallow waters, eutrophication, and the increasing pressure of human-induced changes. Traditional livelihoods, such as fishing, farming, and livestock rearing, have declined in recent decades, as the number of residents (UNESCO—MAB 2022), while tourism is growing in the area (Siivonen 2011; Renfors 2021). As a result, the traditional Archipelago Sea landscapes are under transformation. Guiding the growing tourism in an environmentally, socially, culturally, and economically sustainable direction is important. For this reason, in this article we identify, map and assess the ABR’s diverse stakeholders’ landscape values within the three categories of the IPBES Values Typology (instrumental, intrinsic, and relational). Thus, the aim of this study is to operationalize the IPBES Values Typology, and to apply it in a protected area context. The key research questions addressed are:

- How do different stakeholder groups—locals, recreationists, and tourists—value landscapes?
- Are there conflicts or synergies among these values, especially in relation to tourism?

The research focuses on understanding value pluralism to inform sustainable tourism development that balances environmental, social, and cultural needs. Finally, we analyse whether the diverse landscape values of the Archipelago Sea area coexist, are in conflict, or if there is synergy with identified tourism development preferences.

## Materials and methods

### Study area

**Fig. 1:**
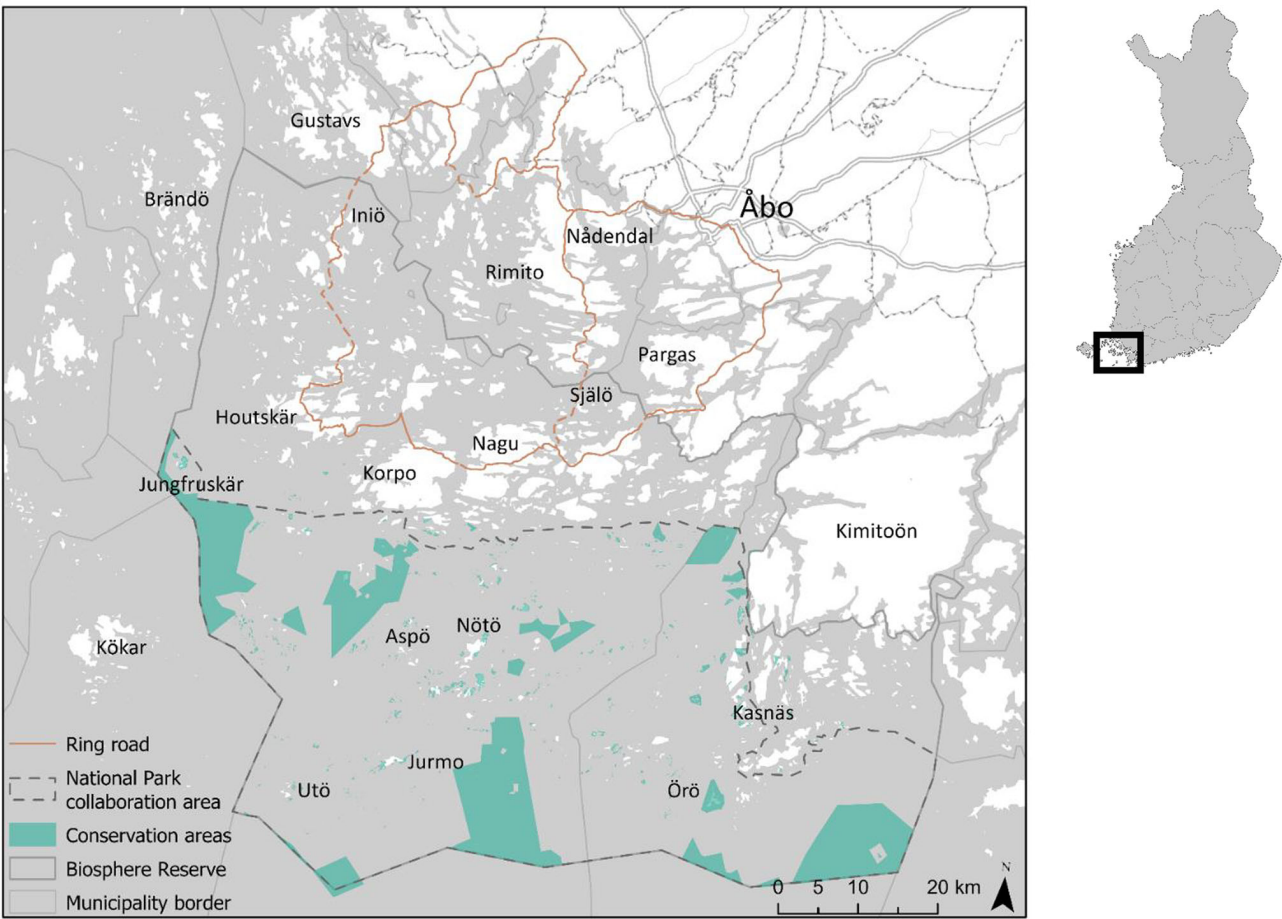
Map of the archipelago sea area

The ABR is situated in Southwestern Finland (Fig. 1). It is the world's most densely populated island concentration, with over 40,000 islands and islets (Renfors 2021) and features diverse habitats like sand and rock islets, meadows, coasts, open sea areas, and uninhabited islands. Humans have played a significant role in shaping the environment for centuries, with farming and grazing creating traditional landscapes that have become ecologically and culturally valuable.

For planning and conservation purposes, the ABR is divided into three areas (Viirret et al. 2019) (Fig. 1). The core zone is the National Park established in 1983. It covers approximately 50 000 hectares including both traditional cultural landscapes and strictly protected areas with minimal human impact (Heinonen 2016; Viirret et al. 2019). The buffer zone surrounding the National Park, comprises privately owned land with moderate human impact (Heinonen 2016; Laurila et al. 2022), including approximately 200 year-round residents and over a thousand vacation homes (Pälvimäki 2020). The transition zone consists of the largest islands with the most significant human impact and land-use pressure (Pälvimäki 2020; Laurila et al. 2022). In total, there are around 3,500 year-round residents living in the different parts of the archipelago (ABR Archipelago Sea Area 2022).

During summer (June–August), activity in the Archipelago Sea Area surges, driven by part-time and summer cottage residents as well as national and international tourists (Heinonen 2016, Viirret). The area offers a plethora of recreational opportunities that showcase its distinctive natural and cultural landscapes. The extensive network of islands and waterways makes the area an ideal location for boating and sailing, offering scenic routes and access to remote areas. The hiking and biking trails traverse a variety of landscapes, including forests, coastlines, and traditional villages, offering visitors panoramic views of the archipelago. Furthermore, birdwatching and wildlife observation are common activities, given that the biosphere is home to a wide variety of bird species and diverse wildlife. Recreational fishing, both freshwater and saltwater, continues alongside traditional practices. Cultural tourism allows visitors to engage with the archipelago's rich heritage through traditional villages, local crafts, and historical sites.

The Archipelago Sea attracts 70 000 + recreationists and tourists annually, mainly during the summer (Pälvimäki 2020). The international appeal of Northern European cold-water tourism is rising due to climate change, while domestic tourism is also on the rise (Sarlin 2009). Since 1996, tourism follows the Archipelago Ring Road (Sarlin 2009), with the majority of tourists choosing these routes. The key difference between small-scale and large-scale tourism lies in their scope. Small-scale tourism involves often locally family run facilities, emphasizing sustainability and minimizing environmental impacts. It aims to benefit local economies through personalized experiences that promote cultural immersion and nature conservation, with examples including eco-lodges and cultural homestays. Large-scale tourism in the context of the Archipelago Sea accommodates more tourists through large hotels or resorts. It offers more standardized landscape and outdoor recreation experiences.

The primary attractions for locals, recreationists and tourists are environmental and nature values, especially those connected to the sea (Renfors 2021). In Finland, everyone's right guarantees that all individuals, irrespective of land ownership, have the freedom to enjoy nature and engage in outdoor recreational activities, while preserving nature, respecting fellow individuals, and safeguarding property (Finnish National Parks 2023).

The Archipelago Sea is the last remaining area in Finland facing nutrient loading issues. Mitigating eutrophication in the sea is a crucial focus and requires reducing agricultural emissions in the catchment area. The 2022 roadmap outlines funding and operational measures to enhance water protection in agriculture (Laurila et al. 2022). Currently, forestry and renewable energy production are also topics that are being developed and discussed regarding the future of the Archipelago Sea area.

### Survey data collection

We collected data through a map-based online survey functioning on the Maptionnaire platform^1^ between June and August 2022. Accessible on various devices and in three languages (Finnish, Swedish and English), the survey comprised seven sections, covering background information, landscape values, and tourism preferences (Supplementary material: Figures S1 to S15). The background information sections asked respondents about their gender, year of birth, education level, home country, occupation, relation with the area, mode of transportation in the area, location when answering the survey, ownership of land in the area, and personal worldviews. The mapping questions began with a test question to show respondents how to map places, after which they were asked to map landscape values and tourism development preferences as point locations. Respondents were asked to map as point features nine landscape values, namely outdoor activity, recreational fishing, hunting and harvest, beautiful landscape or landmarks, wilderness, social relations, cultural history and heritage, biodiversity, and learning and silence. These were chosen based on previous published studies, including Brown and Fagerholm (2015), Fagerholm et al. (2016, 2019), Plieninger et al. (2018, 2021) and Viirret et al. (2019). Furthermore, silence was included in response to a suggestion from the Finnish Forest and Environment Administration (Metsähallitus). Tourism development preferences were also surveyed, which included options for mapping ‘yes’ or ‘no’ for small-scale and large-scale tourism, aligning with prior research (e.g. Plieninger et al. 2018) and input from the Finnish Forest and Environment Administration and the ABR management team. Both organizations were at the time of data collection developing a master plan of the National Park area and the Tourism Road Map. The survey included open-ended questions about respondents' landscape values (e.g. What are the values you most enjoy from the Archipelago Sea Landscapes?), hopes, and concerns related to tourism development (e.g. How do you see the future of Archipelago Sea area? Please share with us 2–3 of your main hopes and/or concerns about the future development of tourism).

The survey targeted full-time residents, recreationists, and tourists, employing a crowdsourced sampling method. Locals are defined as individuals who live, have lived, or work in the Archipelago area. Recreationists include part-time residents and frequent visitors to the area (i.e. more than three times per year). Tourists are national and international and primarily visit the area sporadically for holidays. Survey promotion efforts spanned social media platforms, press releases to local media, and distribution of posters and cards in key public locations throughout the Archipelago Sea area. Additionally, research assistants stationed in the ABR conducted facilitated surveys using tablets in high-traffic locations during June and July 2022, offering small gifts as gestures of appreciation to respondents.

### Analysis

#### Data preparation

Data quality inspection was performed. Distribution of the number of mapped landscape values and tourism development preferences per respondent was checked with a box and whisker plot. For those respondents that had mapped plenty of points and showed as outliers in the box plot (7.8% of respondents mapping landscape values and 15.7% of respondents mapping development preferences), we randomly selected a number of mapped locations that responded the maximum in the distribution. In addition, responses to open survey given in Finnish or Swedish were translated to English by two research assistants.

#### Respondent groups

Respondents were divided into three groups based on how they defined their relationship with the Archipelago Sea area (Table 1): locals (living, having lived or working in the Archipelago area, 171 respondents), recreationists (part-time residents and frequently visitors in their free time, 313 respondents), and tourists (visiting the area, e.g. on holiday, 178 respondents). There were 28 respondents that did not belong to any of these groups and were excluded from further analyses. In terms of survey representativity, the relatively larger share of recreationists compared to locals and tourists raises questions about the balance of perspectives captured. However, it was expected that the recreationists would respond more to the survey as their share of area users is the high. In comparison, the visitor survey of the Archipelago Sea from 2019 showed that 86% of respondents have visited the area previously and only 6% were international tourists (Pälvimäki 2020).

**Table 1:** Survey respondent groups

Respondents group	*n*	*n*%
Locals	171	25.8
Recreationists	313	47.2
Tourists	178	26.8
Total	662	100.0

#### Content analysis

The open question responses were manually coded through inductive content analysis in a data table in Excel. The analysis involved iteratively reading and categorizing similar responses. Categories were identified and presented in Supplementary material (Tables S1 and S2).

Content analysis of landscape values revealed seven different categories related to landscape values that were mentioned a total of 1244 times by 452 respondents (Table S1 in Supplementary material): beautiful landscape or landmark, wilderness and pristine, biodiversity, cultural history or heritage, tranquillity and silence, accessibility, and outdoor recreational activities. These categories overlap with the mapped landscape values, except that accessibility appears as a separate category.

Open responses on tourism hopes and concerns were coded and classified into 9 categories that were mentioned a total of 829 times by 422 respondents (Table S2 in Supplementary material): desired small-scale tourism development, negative environmental impacts of tourism, increasing tourism, nature and local tourism planning, challenges of mobility and accessibility, growing and positive economic impacts, lack of services and products, and support to local communities.

#### Grouping of landscape values according to the IPBES values typology

**Fig. 2:**
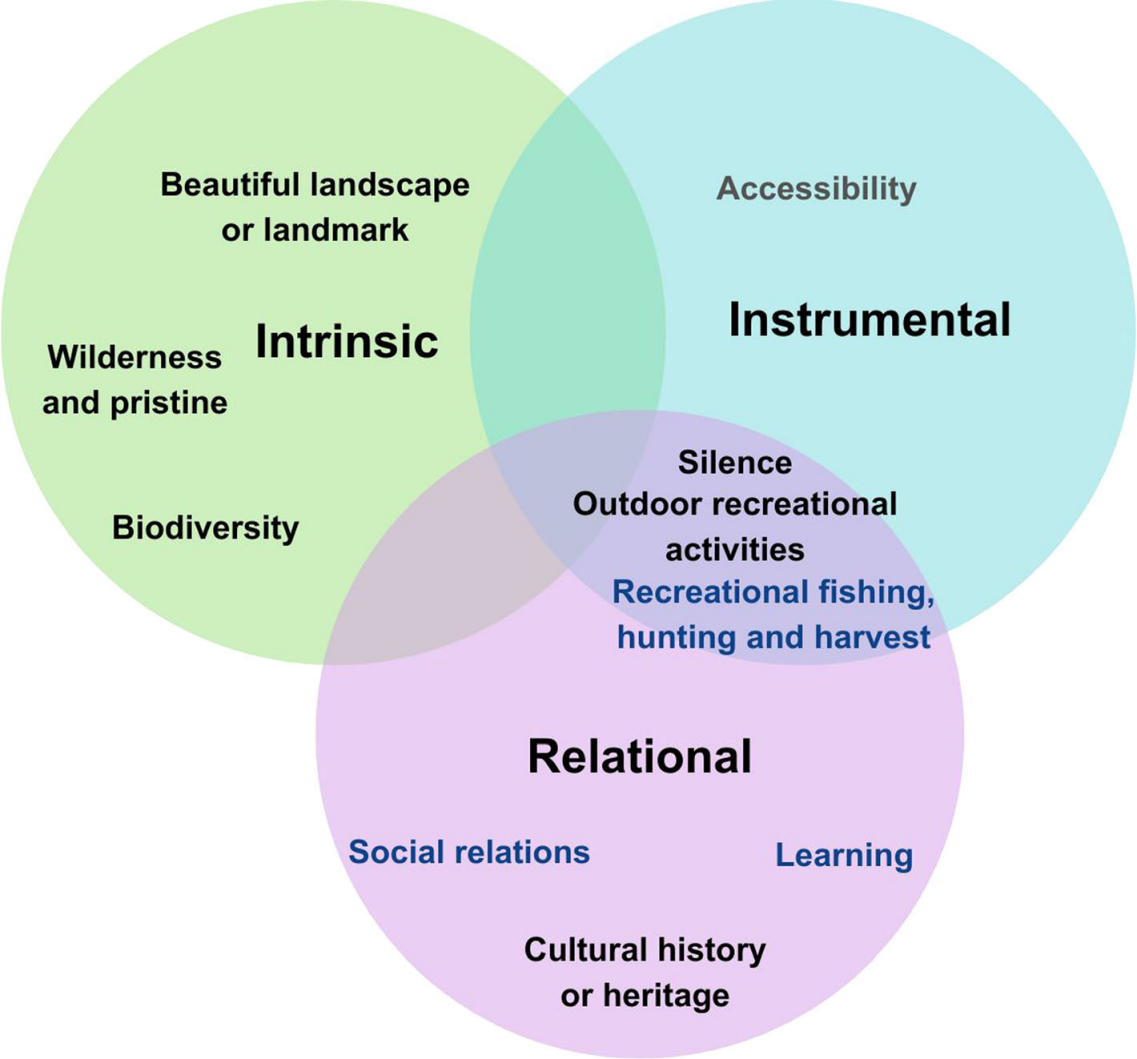
Classification of landscape values that survey respondents identified in the mapping exercise and in the open question according to the intrinsic, relational, and instrumental values presented at the IPBES Values Typology (2022a). The black colour represents landscape values that appear in both the mapping data in the open responses. The blue colour denotes those landscape values that solely appear in mapping questions, while the grey colour denotes a landscape value represented exclusively in the open responses

The nine mapped landscape values and those revealed in the content analysis were categorized based on the IPBES Values Typology (Fig. 2 and Tables S3 and S4 in Supplementary material). Based on our knowledge of the Archipelago Sea area, understanding of the IPBES Values Typology and through iterative discussion among the co-authors, we defined three intrinsic values, three relational values, three relational/instrumental values, and one instrumental value. Respondents frequently identified intrinsic values separately from relational and instrumental values and these are often associated with the unique natural features, biodiversity, and the beauty of the Archipelago Sea (beautiful landscape or landmark, wilderness and pristine, and biodiversity). Values that are exclusively relational (learning, social relations, and cultural history or heritage) referred specifically to the relationships built with nature and through nature. Remaining values are considered both relational and instrumental in the context of the Archipelago Sea (outdoor recreational activities, recreational fishing, hunting and harvest, and silence), connecting landscape with social and cultural meanings intertwined with instrumental values. For example, recreational fishing, was valued for fish as a catch and the human interactions with landscape. Only accessibility, raising from the open responses, was identified as a solely instrumental value because respondents referred to movement opportunities and means to satisfy human mobility preferences.

#### Descriptive statistics

We calculated associations between background variables and respondent groups using descriptive statistics and Chi-square tests in SPSS. We also calculated significant associations between respondent groups and mapped landscape values and tourism development preferences. Likewise, we looked for associations between respondent groups and categories obtained from content analysis on landscape values and tourism hopes and concerns. We used standardized residuals to find the source(s) of the significant association, in cases where we obtained a statistically significant Chi-square result. A residual shows the difference between observed frequency and expected frequency. Standardized residual is beyond the range ± 2, indicate a significant contribution of over- or under-representation of a response. Standardized residuals falling within the range of >  + 2 suggest a weak and non-statistical significance of under- or over-representation of a response (Lane et al. 2017).

#### Spatial analysis

Spatial analysis involved importing respondent-marked points into the ArcGIS Pro 3.0 and a terrestrial data set from the data National Land Survey of Finland (1:250 000) and OpenStreetMap. This dataset set represents the most precise boundary spatial dataset available for the study region. We then clipped this dataset based on the study area boundaries and PPGIS points outside of terrestrial areas were snapped to the nearest coastline based on a 2-km buffer. Only point locations which could be reasonably assigned to a terrestrial location were used.

We developed a new method for creating spatial units which was a compromise between using single islands as spatial units but also gridding larger land masses, in particular contiguous mainland areas, which otherwise would have had a large number of points located on them. Common approaches used in PPGIS analysis for converting point data into a surface, such as using a gridded fishnet (Stahl Olafsson et al. 2022) or density mapping approaches such as Kernel Density Estimation (KDE) (Lechner et al. 2020), are not appropriate for island archipelago study area due to the large expanses of water and combination of large land masses and small islands. Our method facilitated both visualization and analysis grounded in these spatial units.

Using the 20% Quintile of island land size (1108.44 hectares), land masses greater than the maximum spatial unit size were gridded using the subdivide polygon tool in ArcGIS Pro 3.1. This created a map with spatial units made up of single small island spatial units and subdivided larger land masses. Next for each spatial unit we summed the number of PPGIS points for each IPBES Values Typology: intrinsic, relational, and instrumental/relational separately for each of the three respondent groups (see 2.3.5 for explanation on grouping of these value categories). Tourism Development preferences maps were also created (yes to small-scale tourism, no to small-scale tourism, yes to large-scale tourism and no to large-scale tourism) for each respondent groups. Finally, 12 IPBES Values and 16 tourism development preferences choropleth maps were then produced using the Geopandas package in Python. These maps were visually examined to assess coincidences or mismatches between spatial elements, IPBES Values and tourism development preferences. Spatial coincidences indicate that the studied spatial objects align or coincide with each other in space, while spatial mismatches refer to inconsistencies or non-matching spatial objects in the study area.

We also performed a bivariate correlation analysis to examine the relationship between the four IPBES Values and the four tourism development preferences for all three groups. Spearman’s correlation coefficient (*r*) was calculated using Python. In addition, Principal Component Analysis (PCA) biplots were constructed to visualize the relationships between variables and observations in our dataset. The PCA was constructed using the prcomp() function and visualized using ggplot2 in R. These plots can be found in Supplementary Material Figs. S18 and S19.

## Results

### Characteristics of the respondents

In total, 662 respondents participated in the survey. Women responded more frequently than men (*X*2 = 0.330, *p* = 0.847) (Table 2). Most of the respondents were between 25 and 65 years old, with significant differences between respondent groups (*X*2 = 59.806, *p* = < 0.001). Among the tourist respondents, a higher proportion belonged to the age group of 25–44 (44.4%), whereas locals and recreationists tended to be older (45–65 years old). In terms of education, over half of the respondents had a university degree (*X*2 = 6.939, *p* = 0.326). Furthermore, over 90% of the respondents permanently reside in Finland. Respondents’ mobility shows statistically significant differences among respondent groups (*X*2 = 67.530, *p* = < 0.001). Locals have a higher preference for bicycles, while recreationists prefer motorboats and sailboats. Tourists tend to use cars and motorboats more compared to the other groups.

**Table 2:** Relative shares (%) and statistically significant differences in the background characteristics of the respondents divided to the three respondent groups of locals, recreationists, and tourists. Standardized residual beyond the range ± 2 are indicated with bolding. *Respondents could choose several options if they wanted. A total of 588 people answered the question. ***Statistically significant difference between categories (*p* < 0.001)

Gender *X*2 = 0.330, *p* = 0.847 (only female and male)	All (*n* = 662, 100.0%)	Local (*n* = 171, 24.8%)	Recreationist (*n* = 313, 45.4%)	Tourists (*n* = 178, 25.4%)
	*n*%	*n*%	*n*%	*n*%
	(*n* = 534)	(*n* = 136)	(*n* = 244)	(*n* = 154)
Woman	55.2	54.4	55.3	55.8
Man	42.5	43.4	43.9	39.6
Non-binary	0.6	0.7	0.4	0.6
Prefer not to answer	1.7	1.5	0.4	3.9

Age *X*2 = 59.806, *p* = < 0.001***	(*n* = 450)	(*n* = 121)	(*n* = 205)	(*n* = 124)
Under 25	12.7	11.6	**4.9**	**26.6**
25–44	33.3	24.8	31.7	**44.4**
45–65	41.6	49.6	50.2	**19.4**
Over 65	12.4	14.0	13.2	9.7

Education *X*2 = 6.939, *p* = 0.326	(*n* = 552)	(*n* = 149)	(*n* = 243)	(*n* = 130)
Higher education (includes PhD)	64.9	63.8	68.3	60.0
High school/Vocational training	31.2	31.5	28.8	35.4
Basic education (primary and no formal education)	3.8	4.7	2.9	4.6

Mobility* X2 = 67.530, *p* = < 0.001***	*n* = 1242	*n* = 434	*n* = 579	*n* = 229
Car	35.3	31.6	33.3	**47.2**
Motorboat	21.3	21.4	**26.9**	**7.0**
Public transport	17.4	18.2	15.0	21.8
Sailboat	6.5	4.1	**9.0**	4.8
Bicycle	11.7	**15.2**	9.0	11.8
Other	7.8	9.4	6.7	7.4

### IPBES values and differences across respondent groups

#### General patterns

Overall, across all respondent groups, intrinsic values have the highest frequency of mapped sites (52.9%), followed by relational/instrumental (24.3%), and last relational values (22.8%) (Table 3). Remarkably, when the respondents described landscape values in their own words, intrinsic values (77.4%) were even more dominating. Overall, the results show some significant differences in the distribution of IPBES Values categories across the respondent groups, both for the mapped values (*×* 2 = 15.465, *p* = 0.004) and for the open responses (*×* 2 = 15.678, *p* = 0.016) (Table 3), discussed below in more detail.

**Table 3:** Frequency and relative share (%) of intrinsic, instrumental/relational, and relational values. Standardized residual beyond the range ± 2 are indicated with bolding. **Statistically significant difference between categories (*p* < 0.01)

IPBES Values category (map markings) × 2 = 13.692, *p* = 0.008**	All (*n* = 572)		Locals(*n*= 148)		Recreationist (*n* = 268)		Tourists (*n* = 156)	
	freq	%	freq	%	freq	%	freq	%
Intrinsic	1661	52.9	542	55.9	796	50.3	323	55.2
Relational	715	22.8	212	21.9	363	22.9	140	23.9
Relational/instrumental	761	24.3	215	22.2	424	**26.8**	122	20.9
Total	3137	100.0	969	100.0	1583	100.0	585	100.0

IPBES Values category (open responses) × 2 = 15.678, *p* = 0.016**	All (452)		Locals (*n* = 114)		Recreationist (*n* = 220)		Tourists (*n* = 118)	
	freq	%	freq	%	freq	%	freq	%
Intrinsic	963	77.4	249	73.0	495	78.7	219	79.9
Relational	118	9.5	47	**13.8**	45	7.2	26	9.5
Relational/instrumental	137	11.0	41	12.0	73	11.6	23	8.4
Instrumental	26	2.1	4	1.2	16	2.5	6	2.2
Total	1244	100.0	341	100.0	629	100.0	274	100.0

#### Intrinsic values

Intrinsic values emphasize the value of nature itself, its beauty, the wilderness and untouched character of the Archipelago Sea nature, its physical elements (sea, cliffs, rocks) and biodiversity. This was expressed by respondents through quotes such as:

“The sea and beautiful landscapes and their preservation as habitats for animals.” (recreationist male, 36)”

“Beauty, austerity, the archipelago itself, the sea, islets, in earlier times” (tourist female, 76).

In all the groups, the majority of respondents mapped (locals at 50.3%, tourists at 55.2% and recreationists at 55.9%) or described in open responses (locals 73.0%, recreationists 78.7% and tourists 79.9%) intrinsic values (Supplementary Material: Figures S16 and S17). There were no significant differences among the groups.

**Fig. 3:**
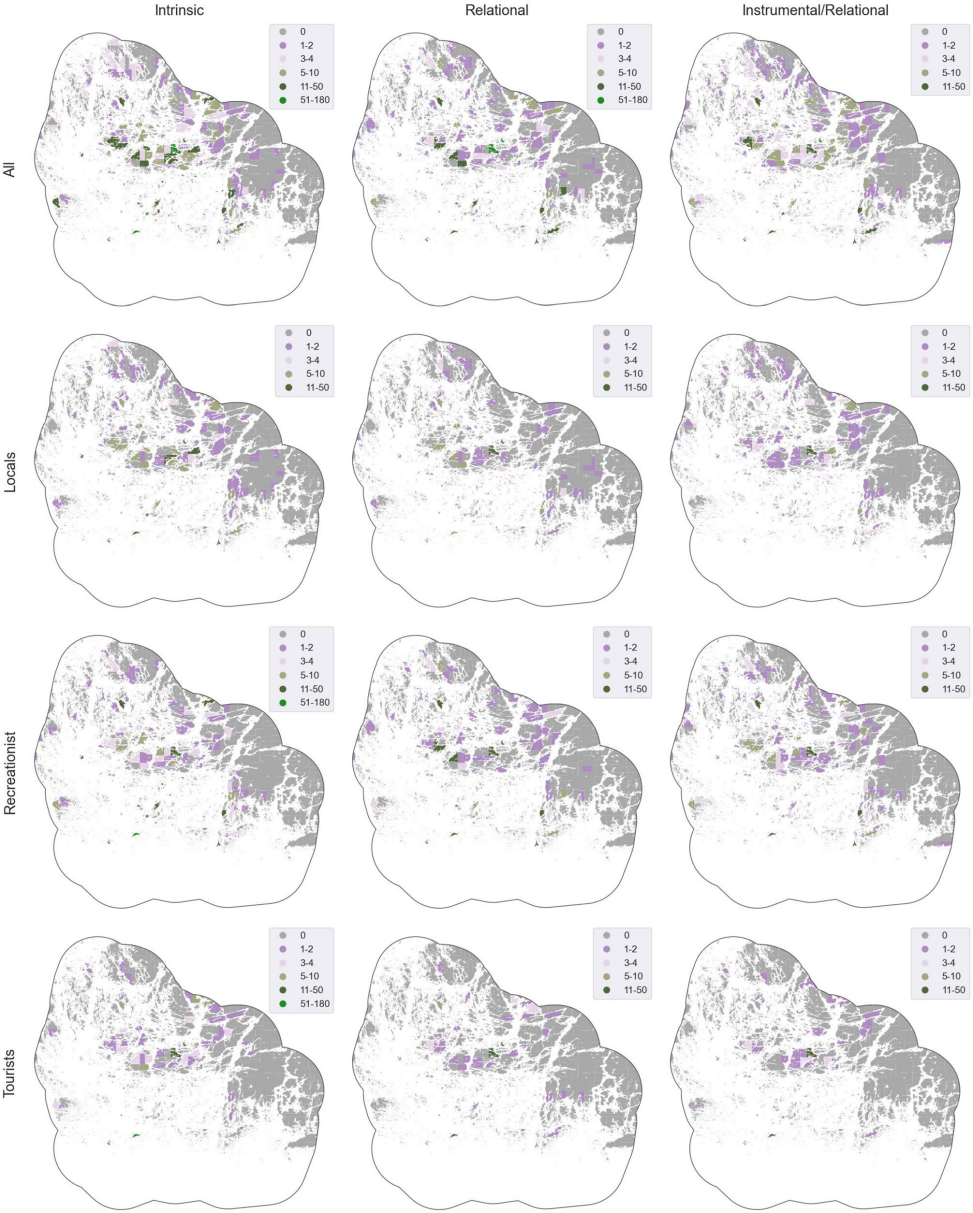
Spatial distribution of the IPBES Values in the whole data and across respondent groups (*n* = 572, 3137 locations) calculated as summed number of points per spatial unit. The study area boundaries were adjusted to align with the nearest coastline, utilizing a 2-km buffer. Data © National Land Survey of Finland and OpenStreetMap

High densities of mapped intrinsic values were observed in areas characterized by picturesque sea, cliffs scenery and traditional villages of the Archipelago Sea (Fig. 3). Sites mapped by locals and recreationists are more dispersed throughout the area, with a higher concentration of marked locations within the National Park. Tourists mapped more sites along the Ring Road and the outer archipelago islands along the ferry route, such as Utö or Jurmo.

#### Relational

The relational value category refers to the relations with nature and through nature that respondents describe, i.e. the importance of traditional livelihoods connected to nature, the harmony with nature, the culture and identity of the area. This was expressed by respondents through quotes such as:

“Living nature, preservation of diverse and sensitive nature and respect for nature, and silence” (local female, 31).

“Calmness, silence, Swedish language, real life with and in the nature” (tourist female, 62)”

There were no significant differences across groups when mapping relational values. However, locals placed more emphasis on relational values when describing these in their own words (13.8% of locals), showing an over-representation of locals in the standardized residuals analysis.

Locals and tourist mapped sites are concentrated in more settled areas, such as larger islands (Nagu, Houtskär, Korpo, Iniö), tourist main attractions (Örö, Själö, Kasnäs) and on the outer archipelago main islands (Aspö,Utö, Jurmo) (Fig. 3), particularly for locals and tourists. Recreationist marked more relational values spread along the National Park area.

#### Relational/instrumental

Relational/instrumental values refer to the beauty of the landscape, the tranquillity and peace, as well as the authenticity and uniqueness of the area. This was expressed by respondents through quotes such as:

“The most important thing in the landscape is the sea and the islands, which shores are not built full of cottages. Especially the outer Archipelago is enchanting because it is more in a natural state and there are many uninhabited islets, etc. Authentic archipelago villages such as Aspö and Utö are also valuable. I think it is important to guarantee year-round living so that the island remain inhabited. Meeting the locals is wonderful and creates new meanings for the Archipelago beyond just functioning as a tourist area.” (recreationist female, 23).

Recreationists mapped significantly more relational/instrumental values (26.8%), showing an over-representation of recreationists in the standardized residuals analysis. In the open responses no significant differences are found between the groups.

In the case of Relational/instrumental recreationists map markings are concentrated in the Natural Park Area, including islands like Jurmo, Utö, Aspö, and Nötö, as well as natural areas surrounding bigger islands, such as Houtskär and Nagu (Fig. 3). Locals show higher concentrations of Relational/instrumental values in settlement areas, as around main villages (e.g. Pargas, Nagu, Houtskär, and Iniö), harbours and touristic hotspots (Kasnäs, Öro, and Sjalö). Tourists focused their place markings primarily on Nagu island, especially around the main village and harbour, and touristic hotspots, such as Själö, Jurmo, Utö and Örö.

#### Instrumental

As the only purely instrumental value, accessibility, respondents particularly remarked on boat routes and freedom of movement referring to “everyone’s right”, for example:

“Access to simple mooring of boat (docks, buoys), well managed walking trails, toilets” (recreationist male, 45).

“Diversity, possibility to travel by water, waterflow, clean sea” (recreationist female, 53).

“Beaches without cottages, where you could land with everyone’s right and a suitable boat (…)” (male, over 65).

In terms of instrumental values, the shares vary across 1.2% (locals) to 2.2% (recreationists) between the groups and are not statistically significant.

### Tourism development preferences and differences across respondent groups

Out of 691 respondents, 61.2% (*n* = 422) shared their opinions on hopes and concern related to tourism development (Table 4). However, based on the Chi-square test, there are no significant differences across the respondent groups (× 2 = 13.324, p = 0.038*).

**Table 4:** Mapped tourism development preferences. Standardized residual beyond the range ± 2 are indicated with bolding. *Statistically significant difference between categories (*p* < 0.05)

Tourism development preferences (map markings) × 2 = 13.324, *p* = 0.038*	All (n = 349)		Locals (n = 93)		Recreationists (n = 163)		Tourists (n = 93)	
	(n = 628)		(n = 177)		(n = 291)		(n = 160)	
	freq	%	freq	%	freq	%	freq	%
Yes to small-scale tourism	301	47.9	77	43.5	148	50.9	76	47.5
No to small-scale tourism	24	3.8	11	6.2	9	3.1	4	2.5
Yes large-scale tourism	97	15.4	38	**21.5**	40	13.7	19	11.9
No large-scale tourism	206	32.8	51	28.8	94	32.3	61	38.1
Total	628	100	177	100.0	291	100.0	160	100.0

Small-scale tourism is generally preferred by all respondent groups (with moderately high percentages ranging from 50.9 to 43.5% between the different groups), followed by a rejection of large-scale tourism (ranging from 38.1 to 28.8% between the three groups). However, locals were the respondent group that marked more locations to support large-scale tourism development (at 21.5%), showing an over-representation of locals in the standardized residuals analysis (Table 4).

Open responses on hopes and concerns in relation to tourism (Table 5, Table S5) also show that the most desired development for all the groups was small-scale tourism (from 14 to 10.1%). This was expressed by respondents through quotes such as:

“The nature of the Archipelago Sea is delicate and large numbers of tourists spoil it. I hope that tourism remains small scale so that nature preserves its uniqueness(..) (recreationist female, 54)”

**Table 5:** Open responses for tourism development hopes and concerns. *Statistically significant difference between categories (p < 0.05)

Tourism development preferences (open responses) × 2 = 22.202, *p* = 0.075*	All (*n* = 428)		Local (*n* = 105)		Recreationist (*n* = 195)		Tourists (*n* = 122)	
	(*n* = 829)		(*n* = 233)		(*n* = 379)		(*n* = 217)	
	freq	%	freq	%	freq	%	freq	%
Desired small-scale tourism development	193	23.3	27	11.6	53	14.0	22	10.1
Negative environmental impacts of tourism	123	14.8	42	18.0	87	23.0	64	29.5
Increasing tourism	104	12.5	26	11.1	45	12.0	30	13.8
Nature and local tourism planning	102	12.3	32	13.7	46	12.1	26	12.0
Challenges of mobility and accessibility	101	12.2	16	6.9	42	11.1	17	7.8
Growing and positive economic impacts	98	11.8	32	13.7	44	11.6	22	10.1
Lack of services and products	75	9.0	43	18.6	49	12.9	31	14.3
Support to local communities	33	4.0	15	6.4	13	3.4	5	2.3
Total	829	100.0	233	100.0	379	100.0	217	100.0

Negative environmental impacts of tourism, such as waste, shoreline construction, or pollution, are also common concerns among respondents (from 29.5% to 18.0%). For example, one of the respondents described:

“The large masses of people and thus the use of nature and emissions on the environment are worrying” (recreationist female, 33)

Other common concerns are increasing tourism (from 13.8 to 11.1%) and nature and local tourism planning (from 13.7 to 12%). Respondents express their worry on the growing tourism trend and the need to find a balance between nature conservation and tourisms development. For example, one of the respondents mentioned:

“The balance between the diversity of the vulnerable archipelago nature and human beneficial and recreational use. How to protect nature values as tourism increases and develops? Perhaps nature tourism can play a part in this”(local female, 22).

Another tension arose between tourism development and mobility (from 11.1 to 6.9%). In this line, a respondent stated:

“Those who live year-round in the archipelago should always have priority on the ferries over the tourists. We are a family with children and if we drive our children on a hobby to the mainland, we don't know if we can get home on the next ferry even though we live all year round at Rosala (…)” (local male, 38).

Nevertheless, these tensions coexist with perceived positive economic impacts of tourism. This was expressed by quotes, such as:

“Domestic tourism enables keeping the archipelago alive” (local male, age not reported)

**Fig. 4:**
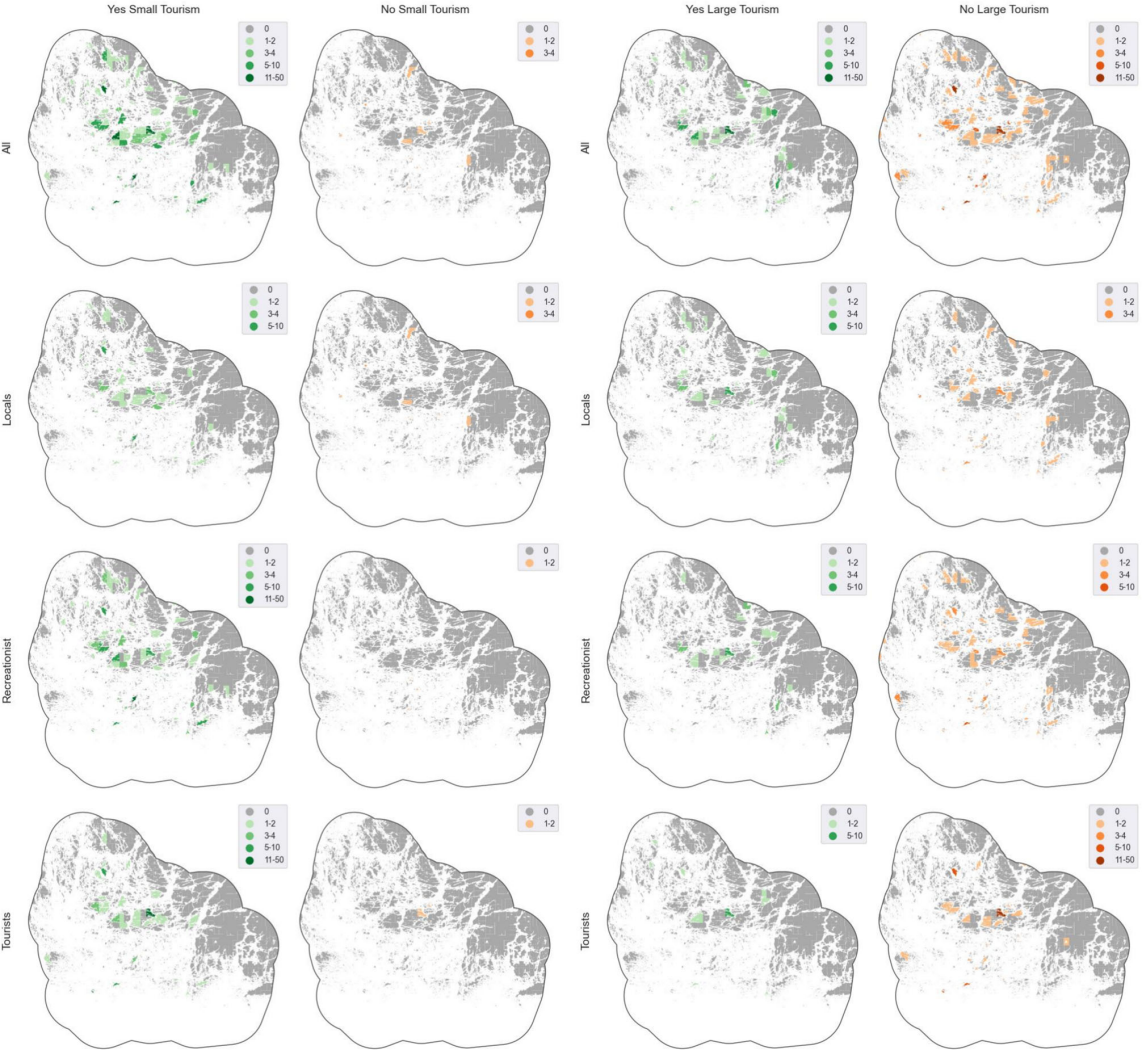
Small-scale and large-scale development reference by groups (*n* = 349, 628 locations) calculated as summed number of points per spatial unit. The study area boundaries were adjusted to align with the nearest coastline, utilizing a 2-km buffer. Data © National Land Survey of Finland and OpenStreetMap

All groups mapped more places to support small-scale tourism of around main villages and harbours in larger islands (Nagu, Korpo, Houtskär, Iniö) and larger outer archipelago islands (Nötö, Jurmo and Utö) (Fig. 4). Locals present higher concentration of mapped places in Iniö and Nötö. Recreationists focus their marking close to main villages in bigger ABR islands, such as Nagu, Korpo, Houtskär and Iniö, and well-known outer ABR islands, as Nötö and Utö. Tourists’ higher concentration marking area in Nagu’s harbour area and Iniö. Additionally, non-small-scale tourism (24 locations/3.8%, Table 4), was the least mapped preference. Locals places these negative responses were located in the Natural Park, as well as in more remote areas within bigger islands, as Nagu, or in the mainland.

Large-scale tourism was generally undesirable in the ABR for all the groups (206 locations/32.8%), particularly in the Archipelago Sea National Park (Fig. 4), but also in larger ABR islands and main villages and harbours (e.g. Nagu, Korpo or Iniö). At the same time, the support to large-scale tourism development was also mapped in these areas. Locals and tourist focused their rejection and acceptance of large-scale tourism development around main villages and harbours of bigger ABR islands. Nevertheless, recreationists tend to place more rejection markings spread across all the ABR and particularly within the National Park, while supporting markings of recreationist for large-scale tourism development are also concentrated around main villages and harbour.

### Relationships among IPBES Values and with tourism development preferences

#### Spatial relationships between IPBES Values across groups

**Fig. 5:**
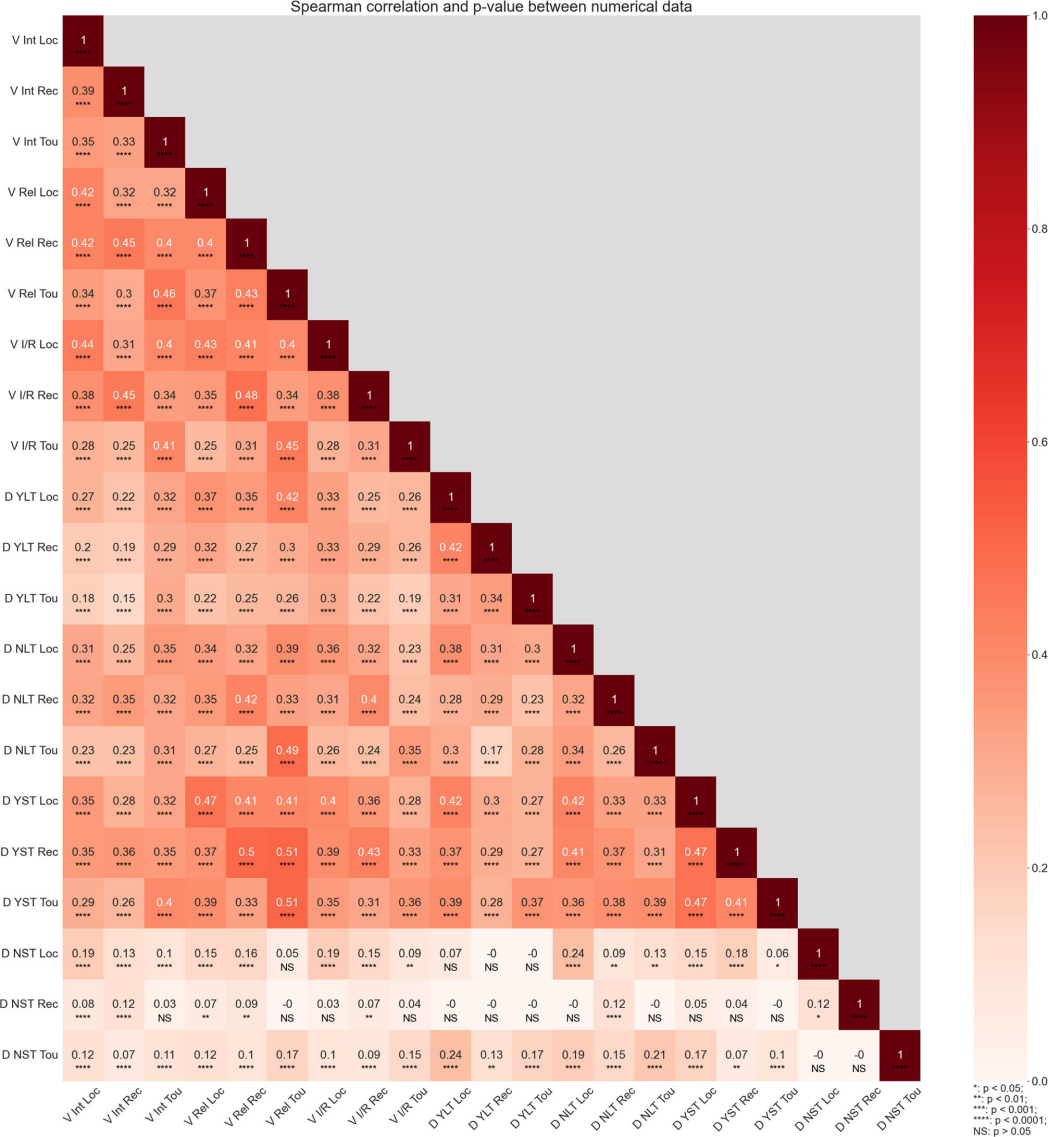
Spatial relationship as expressed by Spearman's rank correlation coefficient (*ρ*) between IPBES Values and tourism development preferences (*n* = 572 IPBES Values, n = 349 tourism development preferences. The correlation coefficient is categorized as a strong correlation when *Rs* ≥ 0.5 (dark red, ***), moderate correlation 0.3 ≤ *Rs* < 0.5 (red, **), weak correlation 0.1 ≤ *Rs* < 0.3 (light red, *) and very weak correlation: 0.09 ≤ Rs ≤ 0.00 (white). V = Values; Int = Intrinsic; REL = Relational. I/R = Instrumental/Relational; YLT = Yes to Large-Scale Tourism; NLT = No to Large-scale Tourism; YST = Yes to Small-scale Tourism; NST = No Small-scale Tourism; LOC = Locals; REC = Recreationists; TOU = Tourists

Our findings reveal moderate spatial correlations (spatial coincidences where one group’s IPBES Values align or coincide with other groups’ IPBES Values within a given area) between the IPBES Values across groups (Fig. 5). For recreationists, moderate correlations can be found for all IPBES Values, with stronger correlations identified between instrumental/relational and relational values. Similar, in the cases of locals and tourists, stronger correlations occurred between instrumental/relational and relational, but also between instrumental/relational and intrinsic values.

Additionally, we observed a few moderate correlations across groups, such as between recreationist relational values and locals' intrinsic values. Likewise, we identified moderate correlations between relational values for recreationists and tourists. Also, the PCA graph shows high loading of the first component (70.42%) with all variables having almost similar positive loadings (Supplementary Material: Figure S18).

#### Spatial relationships between IPBES Values and tourism development preferences across groups

Our findings indicate strong spatial correlations (*p* < 0.05) between IPBES Values and preferences for tourism development (Fig. 5). Spearman's rank correlation analysis revealed few strong correlations but several moderate correlations between these variables.

Among recreationists and tourists, strong correlations were observed between relational values and positive responses to small-scale tourism development. These spatial correlations are evident in the Natural Park Area, encompassing outer archipelago islands like Utö and Nötö, as well as larger islands such as Nagu, Houtskär, and Iniö, particularly around more settled areas.

Moderate correlations were found for recreationists and locals, linking positive responses to small-scale tourism with instrumental/relational values. These spatial correlations occurred in settled areas, notably in Nagu and Korpo. Locals also exhibited a strong moderate correlation between endorsing small-scale tourism and relational values, predominantly observed in Nagu and Korpo islands, particularly in the harbour area, and the outer archipelago islands of Nötö, Jurmo, and Utö.

Negative responses to large-scale tourism were correlated with IPBES Values, such as responses to relational values for both recreationists and tourists. For recreationists, these spatial coincidences are more relevant in the outer archipelago islands and the National Park. For tourists, these spatial coincidences are particularly notable in Nagu and Iniö, especially around the harbour areas, and in well-known outer archipelago islands like Jurmo and Utö.

## Discussion

### The operationalization of IPBES values typology

This paper operationalizes the IPBES Values Typology to assess landscape values across different population segments in the ABR. It highlights the typology's role in supporting socio-cultural valuation and accommodating value diversity. While economic valuation dominated until 2015, recent years have witnessed a notable rise in interdisciplinary and socio-cultural valuation methods (Acharya et al. 2019). Gross et al. (2023) indicate a shift from economic to socio-cultural valuation in protected areas, aligning with international initiatives like IPBES promoting value pluralism and the integration of socio-cultural valuation in landscape management (Díaz et al. 2015). The typology of IPBES Values aims to enable a more thorough recognition of value plurality in complex decision-making processes (Pascual et al. 2023). Our research has shown that the IPBES Values Typology needed to integrate the vagueness, complexity, and context-dependency of people-nature relationships in the ABR. This was revealed especially by our qualitative open survey questions. For example, our results show some differences between mapping and open responses within the locals who may not prioritize place-based relational values but emphasize them in their own descriptions. Hence, we consider that qualitative methods are vital for integrating value plurality in protected area valuation. Also, in ABR context, some landscape values are both relational and instrumental, reflecting the use of nature for human needs while also arising from shared relations with nature. Similarly, Himes et al. (2024) discovered that the boundaries between values are often blurred, with the meanings of instrumental, intrinsic, and relational values overlapping in certain contexts. Moreover, they emphasize how relational and instrumental values intersect, particularly concerning material elements like food. The valuation of these material contributions may shift between primarily instrumental or relational and be contingent upon the specific context and local customs (Himes et al. 2024).

Furthermore, in our open responses, respondents frequently mentioned ambiguous concepts, such as nature, biodiversity, or landscape diversity. These concepts can be conceptualized in multiple ways, but our data do not tell how respondents understand them. The construction of these concepts, such as nature, and by whom it is constructed can play a decisive role in influencing which human-nature interactions, ways of life, cultures, and what values are enhanced or marginalized by policy structures (Willemen et al. 2023). However, this appreciation may not align with dominant conservationist approaches advocating for ‘pristine nature’ conservation, but instead with alternative ways of relating to nature and non-dominant normative positions. These non-dominant views and positions should be integrated into land-use planning. Further research is needed to better understand the diverse meanings attached to nature, biodiversity, and landscape diversity, including how different stakeholders interpret these concepts and the normative positions associated with their understandings (Pascual et al. 2023).

Although the recognition of value pluralism is vital for effective protected area planning, as it acknowledges diverse values held by individuals and communities, the impact of value pluralism studies on decision-making is still limited (IPBES 2022b). To enhance the acceptance of valuation approaches, Barton et al. (2022) and Pascual et al. (2023) suggest adjusting and timing valuation to align with specific policymaking requirements and decision-support opportunities. Incorporating local values, particularly those of traditionally marginalized groups, results in more just and sustainable decision-making outcomes (IPBES 2022a; Pascual et al. 2023). In addition, Zafra-Calvo et al. (2020) argue that through transdisciplinary plural valuation studies, a real impact on decision-making can be achieved.

From its outset, the research presented here was partly embedded in a transdisciplinary process. The survey was co-designed with national and regional administrations (Finnish Forest and Environment Administration and the ABR management team) with both working on planning instruments for the area. The survey data collected addressed the needs of these ongoing planning processes, and the results were analysed and presented to the Finnish Forest and Environment Administration officers in forums where they could comment and make recommendations. Additionally, an open-access report about landscape values and development preferences at the Archipelago Sea (Solé et al. 2023, only available in Finnish), was published, presenting the main results. The long-term impact of this study on decision-making is still uncertain. Further research should explore how planning tools in the area have integrated the plurality of values and how this plurality has translated into real-world impactful actions.

While the tangible implications of this study have yet to be determined, the implementation of a PPGIS methodology offers a number of advantages in elucidating the spatial relationships between IPBES values and tourism development preferences. PPGIS generates spatial data that visualize how different user groups perceive and value landscapes, providing crucial insights into the geographic distribution of preferences and values related to tourism development. Also, PPGIS' simultaneous collection of qualitative and quantitative data allows for a nuanced understanding of landscape values. In addition, PPGIS can identify potential conflicts between different stakeholders' values and tourism preferences and visualize these differences to facilitate discussions and negotiations to find common ground. Incorporating value pluralism and related conflicts into policy development for tourism and environmental management requires an inclusive approach that recognizes and integrates the diverse values held by different stakeholders. This approach ensures that policies reflect not only economic concerns, but also the socio-cultural, relational and intrinsic values associated with natural landscapes and ecosystems. However, integrating our findings into existing decision-making frameworks can prove complex, particularly in bureaucratic settings where established procedures may not easily accommodate new knowledge (Denwood et al. 2022; Kantola et al. 2023). In addition, implementing PPGIS can be resource intensive, requiring significant time, funding and technical expertise that may not be readily available, particularly in smaller communities (Brown and Kyttä 2014; Ramirez et al. 2023).

### Planning the future of the archipelago sea biosphere reserve with value diversity

The results show the importance of intrinsic values and reveal distinct distribution patterns of IPBES values across groups. Locals mapped more intrinsic and relational values, recreationists had higher representation of relational/instrumental values, and tourists showed a preference for intrinsic and relational values. The differences suggest that these groups may have distinct motivations for visiting and engaging with these environments. Recognizing these differences aids in designing more effective management strategies that meet the diverse needs of various groups that recognize conflicting values that are evident in specific locations (Brown and Raymond 2007; Lechner et al. 2020; Lourdes et al. 2024).

For locals, the preservation of natural environments is important, but built-up landscapes within the ABR also hold significant relational values. In this context, there is a spatial correlation between instrumental/relational and intrinsic values identified at the same locations. Finding a balance between preserving natural spaces and accommodating areas with more intense human activities is crucial.

The observed spatial correlation between relational and intrinsic values among tourists indicates that individuals within this group emphasize the social and interpersonal aspects of landscape. Tourists prioritize the relational and intrinsic values, particularly in easily accessible sites that are linked to the ABR traditional local landscape, culture, and heritage. Preserving the heritage and landscapes of the ABR is a key aspect for tourists.

Recreationists view natural areas as important spaces for recreational activities that foster social connections with nature and emphasize the need for infrastructure that minimizes environmental impact. Notably, the strongest spatial correlation is found between instrumental/relational and relational values for recreationists. Moreover, recreationists also show a correlation between instrumental/relational and intrinsic values. These correlations indicate that recreationists, while recognizing the instrumental or utilitarian aspects of the environment, also place a considerable emphasis on relational values and intrinsic values, reflecting a multifaceted appreciation for landscape, considering its utilitarian, relational and intrinsic aspects.

In fact, across all groups, a consistent pattern emerges with stronger correlations between instrumental/relational and relational values, emphasizing the interconnectedness of these dimensions. For instance, activities like fishing, hunting, or harvest were found to be intertwined with relational values, emphasizing the social aspect of these practices. Specifically, locals and tourists demonstrated robust correlations between instrumental/relational and intrinsic values. Examples include connections between silence and perceptions of wilderness and pristine environments.

The focus on both instrumental and relational/intrinsic values among groups underscores the necessity for policies that not only facilitate sustainability through economic growth but also encourage environmental stewardship and enhance the quality of life for local communities. In this regard, tourism policies should incorporate frameworks that recognize not only the economic benefits of nature-based tourism but also the relational and intrinsic values associated with recreational activities. Such an approach could entail the development of indicators that assess these non-monetary values in conjunction with traditional economic metrics. Furthermore, the implementation of policies that monitor the health of ecosystems within areas of nature-based tourism is of paramount importance. This would require monitoring the effects of visitor activity and guaranteeing that natural environments are preserved in a manner that allows for both recreational use and the maintenance of ecological integrity.

### Tourism development preferences and planning

The results highlight variations among respondent groups regarding tourism priorities and concerns. Recreationists prioritize small-scale tourism development, while tourists and recreationists express concerns about environmental impacts and growing tourism challenges. Locals are more concerned about nature, local tourism planning, and community support.

When looking at the spatial correlations between values and tourism development preferences, notable patterns emerged within the recreationist and tourist groups, showing strong correlations between relational values and positive responses to small-scale tourism development, particularly around settled areas in larger and well-known outer archipelago islands. Similarly, examining dynamics with locals reveals moderate correlations linking positive responses to small-scale tourism with instrumental/relational and relational values, also concentrated in settled areas and well-known outer archipelago islands. This suggests locals view these areas as social and functional spaces, holding positive attitudes towards small-scale tourism development in these areas.

Conversely, negative responses to large-scale tourism correlate with IPBES Values, particularly relational values for both recreationists and tourists. Recreationists show pronounced spatial correlations in the outer archipelago islands and the National Park, reflecting reluctance to large-scale tourism in ecologically sensitive areas. Tourists, however, display significant spatial correlations along the ring road and ferry route, indicating their rejection of large-scale tourism development in areas they are familiar with.

Traditional local landscapes, culture, and heritage are highly valued by all respondent groups, representing important aspects of the relational values. However, the ABR is currently facing a significant decline in traditional livelihoods and residents who play a crucial role in shaping and preserving these landscapes and local culture. This poses a fundamental question about preserving traditional landscapes and local heritage while the driving forces that sustain them are disappearing.

Tourism is considered a potential solution, but its development must involve participatory processes to hear and understand the needs and preferences of different actors. Our survey revealed that respondents support small-scale tourism, near main villages and harbours on larger islands and the main outer archipelago islands but oppose it in remote and natural areas. Additionally, the survey showed a general aversion to large-scale tourism across all respondent groups and throughout the Archipelago Sea, particularly within the National Park and collaboration area. This suggests a preference for limited and controlled tourism development, focusing on smaller-scale operations in specific areas, while discouraging extensive large-scale tourism, especially in natural and protected areas.

Our results highlighted concerns about the environmental impacts of tourism and the necessity for sustainable planning across all respondent groups. Locals emphasized nature conservation and tourism planning. Similar sentiments were found in a PPGIS survey on Tioman Island (Malaysia) by (Lechner et al. 2020), where various respondent groups strongly supported tourism management and conservation efforts. Given the unique features of islands as tourist destinations—characterized by vulnerability, insularity, and peripherality—they will face increasing pressure due to the cumulative impacts of economic, social, and environmental changes (Lim and Cooper 2009; Carlsen and Butler 2011; Renfors 2021). Consequently, effective planning and management strategies that balance tourism development with nature protection are fundamental, particularly for islands emerging as tourist destinations.

In the Nordic region, nature-based tourism has been growing in the last decade, especially in rural and peripheral areas (Fredman and Tyrväinen 2010, Øian et al. 2018, Fredman et al. 2021). It is advocated as a mutually beneficial solution that can address the inherent conflict between tourism growth and limited natural resources (Duffy 2015). The ABR is also an attractive cold-water tourism destination when the climate change impacts become even more severe in central Europe and elsewhere. There is not a unique and universal definition of nature-based tourism (Tyrväinen et al. 2014), but it can be defined as tourism located in natural areas and with opportunities for individuals to partake in a diverse range of outdoor activities connected to nature (Hall and Boyd 2005; Hall 2009; Lundmark and Müller 2010). Biosphere Reserves have seen a significant increase of nature-based tourism (Ryan et al. 2013). The Biosphere Reserve program aims to enhance the harmony between nature and people and hence to become “learning places for sustainable development” (UNESCO 2023). This vision seeks to make the Biosphere Reserves, such as the ABR, places where human activities, particularly increasing tourism development, coexist in harmony with traditional landscapes, cultural heritage, and nature protection.

Nature-based tourism can positively impact individual and collective well-being, contributing to sustainability (Winter et al. 2020), engaging in outdoor activities, visiting natural attractions, developing a connection with nature, supporting environmentally responsible behaviours (Cartwright 2017; Otto and Pensini 2017), and improving mental and physical health (Mayer et al. 2009). Additionally, nature-based tourism has the potential to enhance community resilience through skill development and economic diversification (Lew and Cheer 2017). However, nature-based tourism also poses challenges in peripheral areas of Biosphere Reserves where local communities are more vulnerable to external impacts (Hall and Boyd 2005). Rapid, and uncontrolled growth can generate conflicts between visitors and local communities for competition for resources and infrastructure, or over benefits distribution (Andereck et al. 2005; Bricker et al. 2010), making tourism development in Biosphere Reserves a double edge weapon.

In this context, Dabard (2024) studied the transformative potential of sustainability innovations in Biosphere Reserves. Their results show the importance of relational values for tourism entrepreneurs as a means to create and promote new services and products that enhance sustainability transformations. Our study aids in determining the values present in the area but to also learn from them to lay the groundwork for future tourism management and policy solutions. Our relationship with nature and through nature should be a guiding principle of Biosphere Reserves' tourism management to align with the Biosphere Reserves' main goal of coexistence with nature. Relational values should not be only used to create new services and products, but to better understand the complexity of human-nature relations in Biosphere Reserves and to support their management towards sustainability.

We consider that the nature-based tourism sector has the potential to capitalize on relational values by promoting experiential forms of tourism, such as guided nature walks, cultural experiences, and community-based services. This approach has the potential to enhance the visitor experience while supporting local economies. An understanding of the values that local communities ascribe to their environment can inform the development of tourism offerings that align with local heritage and culture, while also facilitating more profound connections between tourists and the environment.

## Conclusions

The implementation of the IPBES Values Typology in the ABR reveals the diverse landscape values held by different population segments in alignment with international initiatives like IPBES. While the IPBES Values Typology aims to identify the diversity of values in complex decision-making processes, in our analysis we found the typology needed to acknowledge the vagueness, complexity, and context-dependency of people-nature relationships in the ABR. Nevertheless, our approach supports inclusive decision-making, reinforcing this shift, and deepens our understanding of human-nature relationships. Moreover, this empirical research serves as a model for holistic valuation of protected areas like Biosphere Reserves. The findings can inform sustainable development strategies that consider diverse stakeholder values and preferences, leading to more effective landscape management that balances environmental, social, and economic needs. However, translating value pluralism into decision-making remains limited and further research is needed here.

The findings of this study emphasize the importance of intrinsic values and the varied distribution of IPBES Values among different stakeholder groups. Additionally, the strong correlations between values, such as instrumental/relational and relational values, highlight the interconnectedness of these dimensions, showcasing the complex appreciation of landscapes.

The study also reveals variations in tourism priorities and concerns among different respondent groups, emphasizing the need for participatory processes in tourism development planning. The preference for small-scale tourism development in specific areas, coupled with aversion to large-scale tourism, reflects a collective desire for sustainable tourism practices that preserve nature and local heritage. Nature-based tourism emerges as a potential solution, offering opportunities for economic development while fostering environmental conservation and community resilience.

## Supplementary Information

Below is the link to the electronic supplementary material.

Supplementary file1 (PDF 2236 KB)

## Data Availability

The survey data are available at the University of Turku Geospatial Data Service at: https://geonode.utu.fi/catalogue/#/document/369.

## Footnotes

1 https://new.maptionnaire.com/q/4bky4vfe7y97.
