# Supplementary material for: Balancing landscape values and tourism choices: Integrating participatory mapping and the IPBES Values Typology
Source: Ambio. 2025 Jan 3;54(5):818–38. doi: 10.1007/s13280-024-02112-6 (PMC11965060; doi:10.1007/s13280-024-02112-6)
Supplement: Supplementary file 1 — Supplementary file1 (PDF 2236 KB) [file 13280_2024_2112_MOESM1_ESM.pdf]

Supplementary Information

Balancing Landscape Values and Tourism Choices: Integrating Participatory Mapping and the IPBES Values Typology

Solé, L.[a,b], Hearn, K.P.[c], Witra, T.[d], Lechner, M. A.[d], Fagerholm, N.[a*]

[a] *Department of Geography and Geology, University of Turku, 20014 Turku, Finland*
[b] *Department of Geography, Universitat Autònoma de Barcelona, Spain*
[c] *Department of Human Sciences and Education, Universidad Pública de Navarra, Pamplona, Spain*
[d] *Monash University Indonesia, Green Office Park 9, The Breeze BSD City, Tangerang Selatan, Banten 15345, Indonesia*

*Corresponding author. Nora Fagerholm. ncfage@utu.fi

Supplementary Information

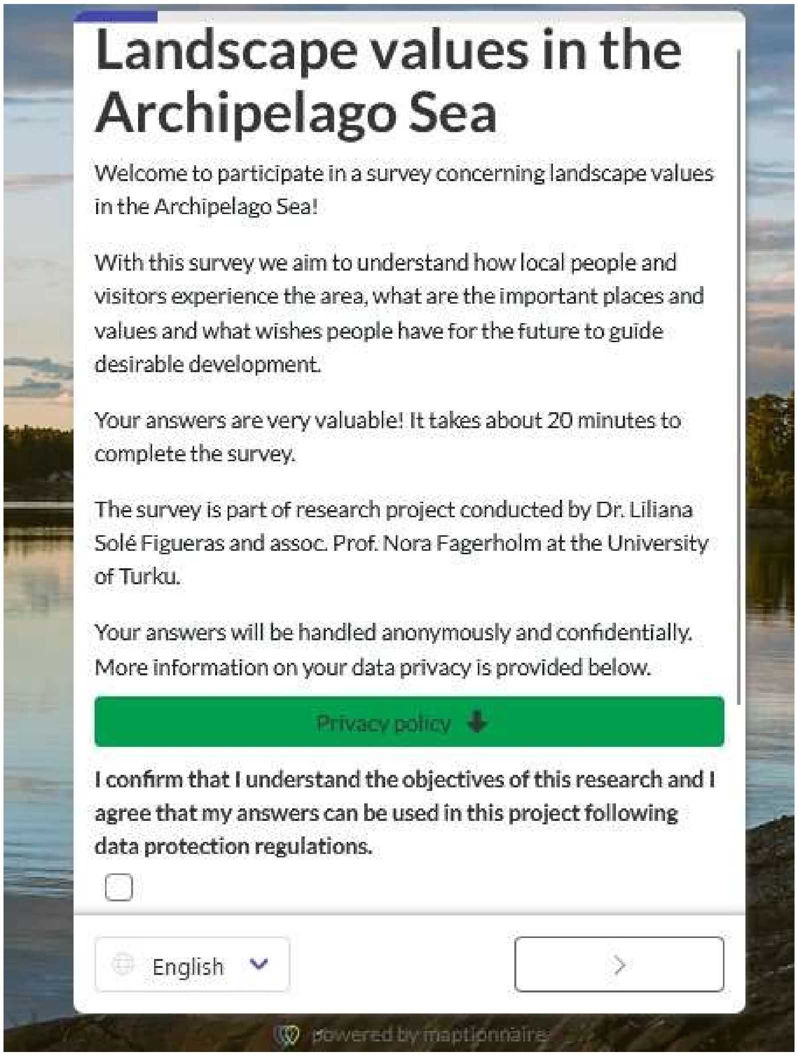

Figure S1. The introductory page of the survey described the survey and its implementation, as well as the parties responsible for the research project. The survey was available in Finnish, Swedish and English.

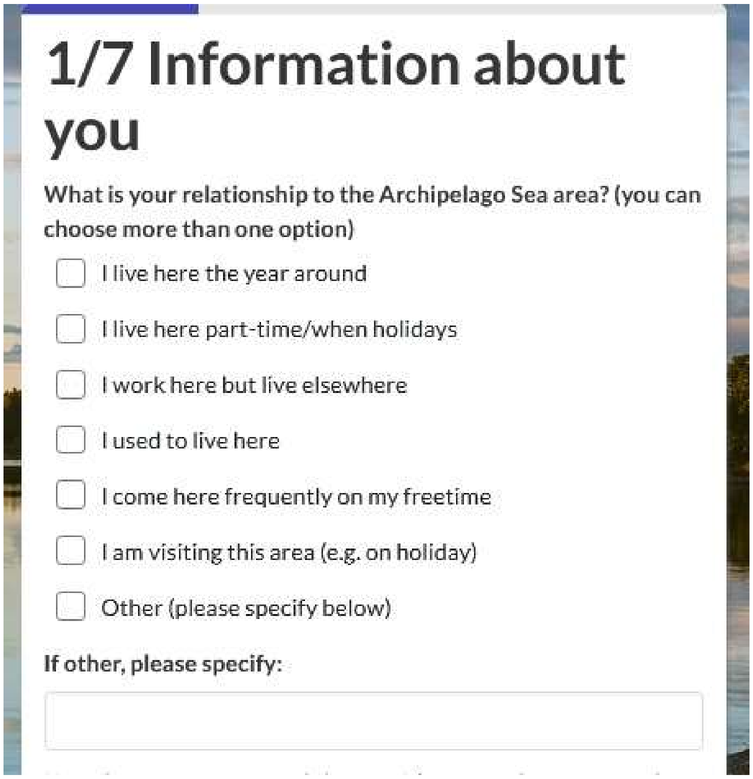

1/7 Information about you

What is your relationship to the Archipelago Sea area? (you can choose more than one option)

- [ ] I live here the year around
- [ ] I live here part-time/when holidays
- [ ] I work here but live elsewhere
- [ ] I used to live here
- [ ] I come here frequently on my freetime
- [ ] I am visiting this area (e.g. on holiday)
- [ ] Other (please specify below)

If other, please specify:

Figure S2. The first section asked about the respondent's background information, such as the relationship with the Archipelago area.

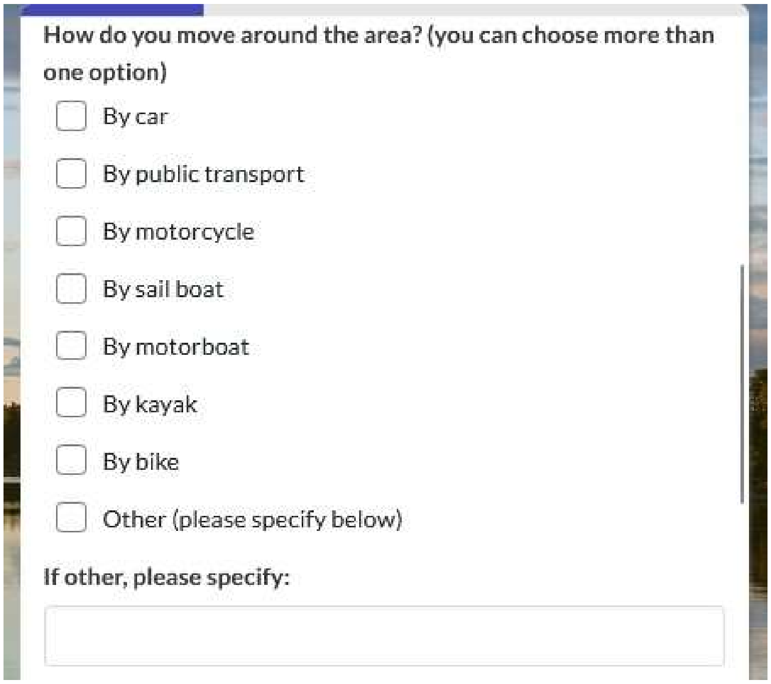

How do you move around the area? (you can choose more than one option)

- [ ] By car
- [ ] By public transport
- [ ] By motorcycle
- [ ] By sail boat
- [ ] By motorboat
- [ ] By kayak
- [ ] By bike
- [ ] Other (please specify below)

If other, please specify:

Figure S3. On the first page, the respondent had to answer the ways of moving around the area.

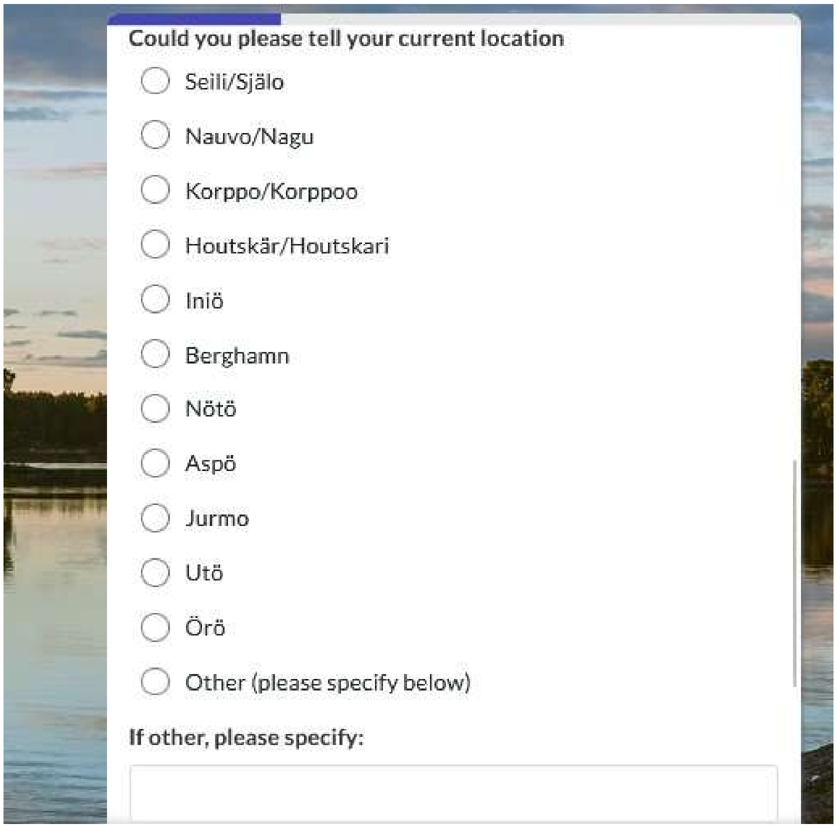

Figure S4. Respondents were also asked in the first page about their location when answering the survey.

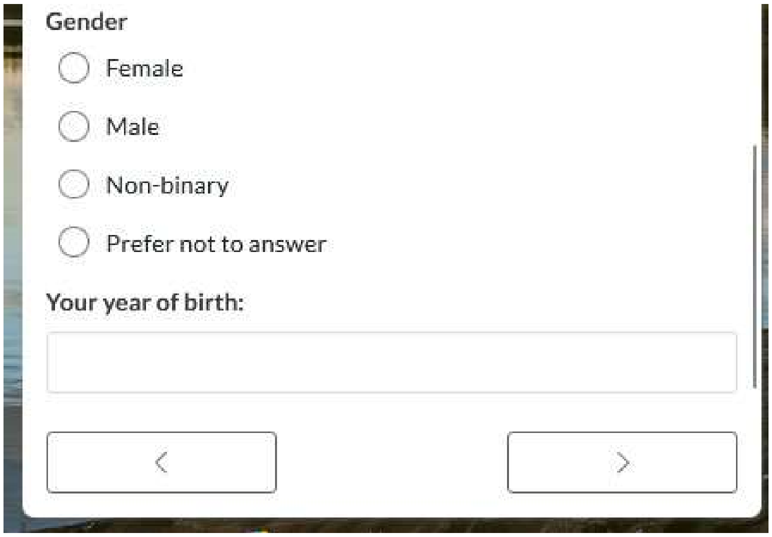

Figure S5. At the end of the first page, the respondent's gender and year of birth were asked.

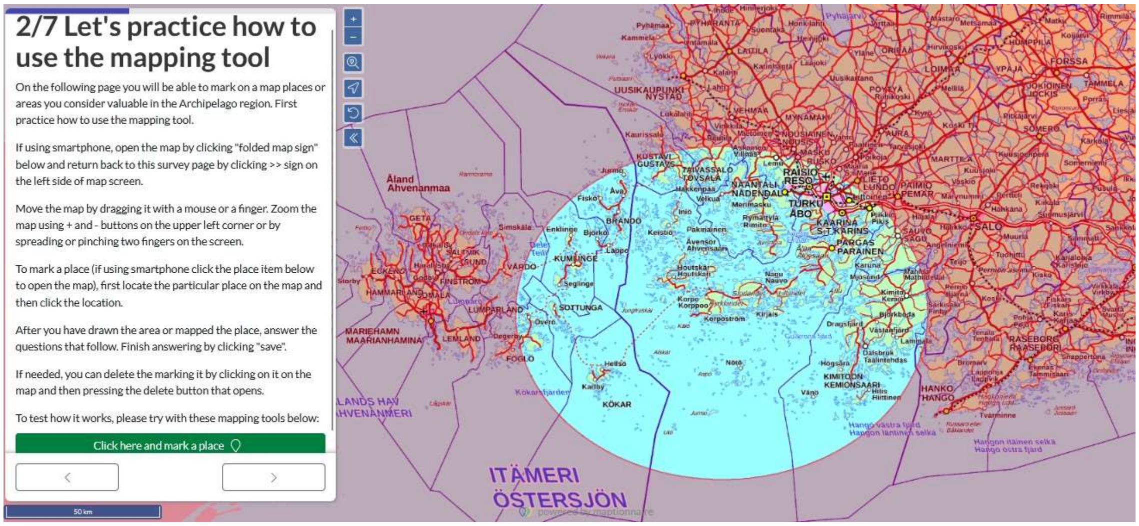

Figure S6. In the second section, the respondent could practice making map markings.

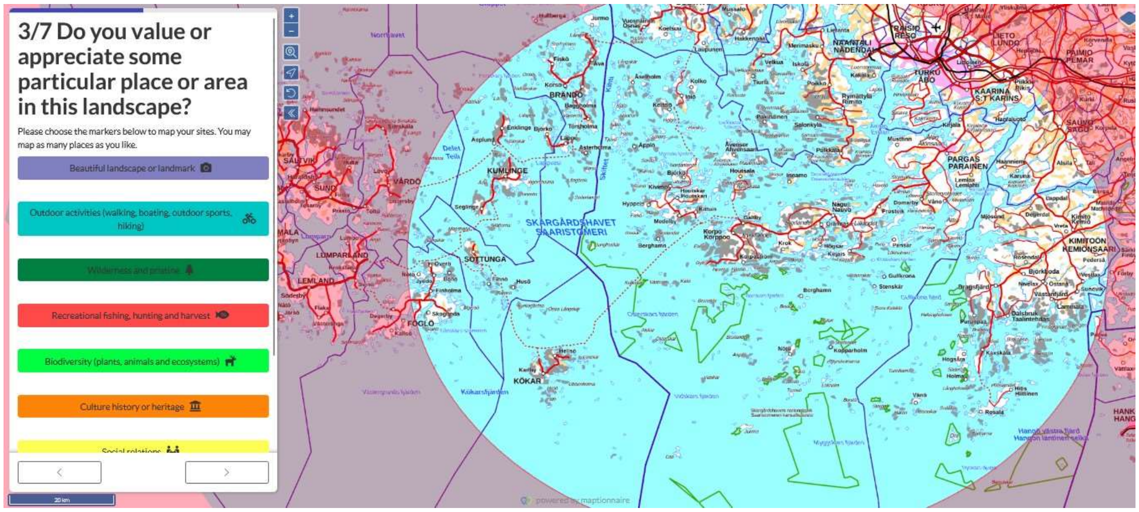

Figure S7. In the third section, the respondents were asked to map places that are valuable or important to them. The ready-defined answer options (see table A1 of this appendix) described different landscape values.

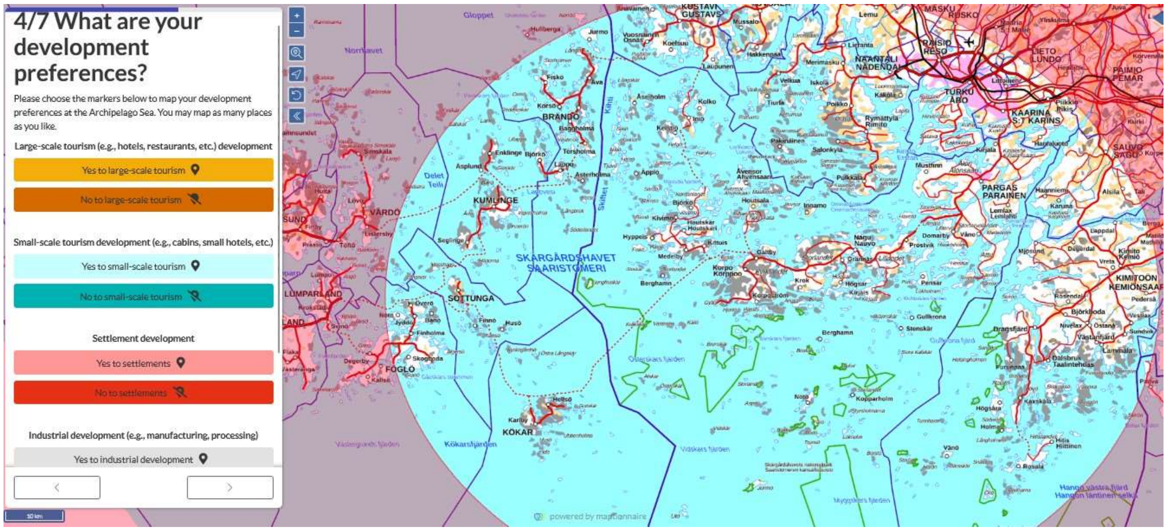

Figure S8. In the fourth section of the survey, the respondents could mark different development preferences on the map. The possible answer options were: yes to small-scale tourism, no to small-scale tourism, yes to new settlement, no to yes to new settlement, yes to industrial activity, no to industrial activity, yes to wind power energy development, no to yes to wind power energy development (see table 2 of the report). The respondents were allowed to mark on the map the locations that they consider the activity is suitable or not. In this article, the focus is only on the tourism development preferences.

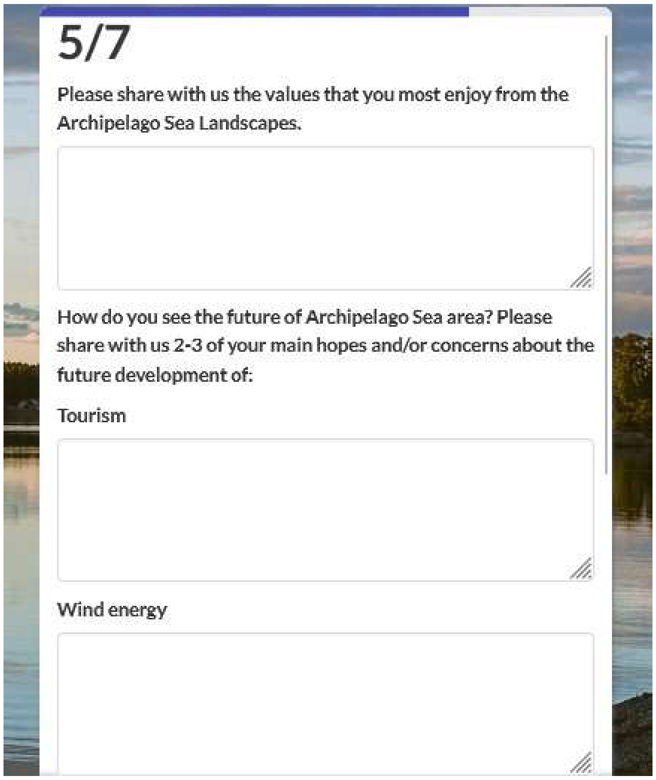

Figure S9. The fifth section asked what is most valuable to the respondent in the landscape of the Archipelago Sea. In addition, wishes and/or concerns were asked regarding tourism, wind power,

eutrophication and climate change. In this article, the focus is only on wishes and/or concerns related to tourism.

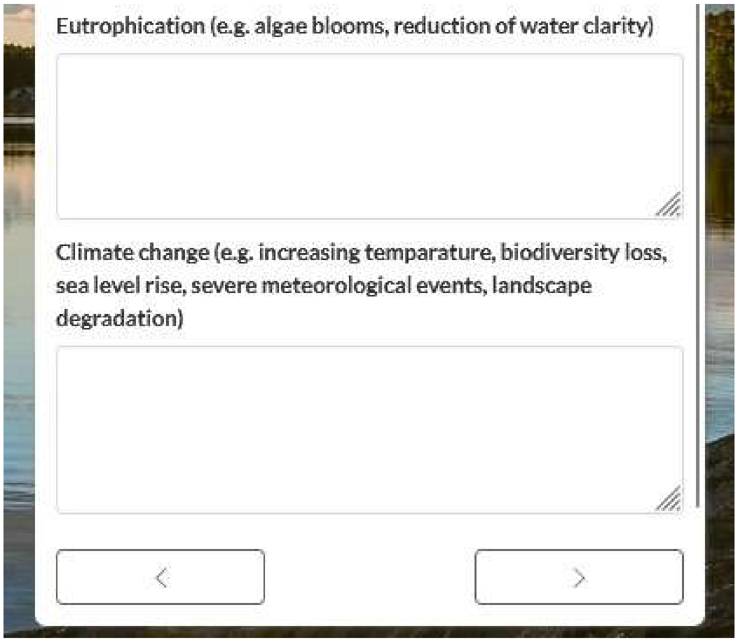

Figure S10. The rest of the fifth section

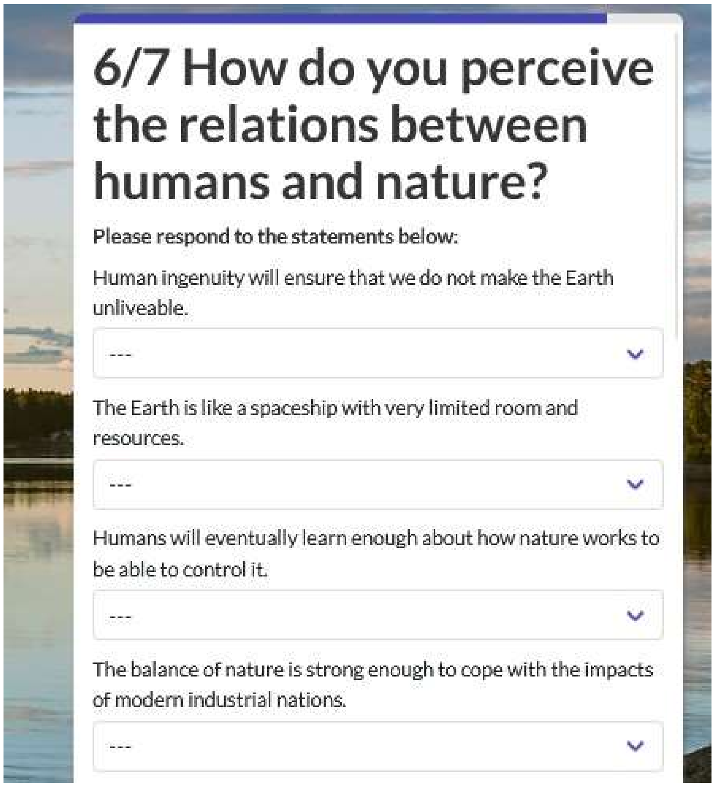

Figure S11. In the sixth section, respondents were asked about their opinion on the relationship between man and nature. They were asked to answer rate statements, answered, such as whether man is meant to control nature. These are not treated in this article.

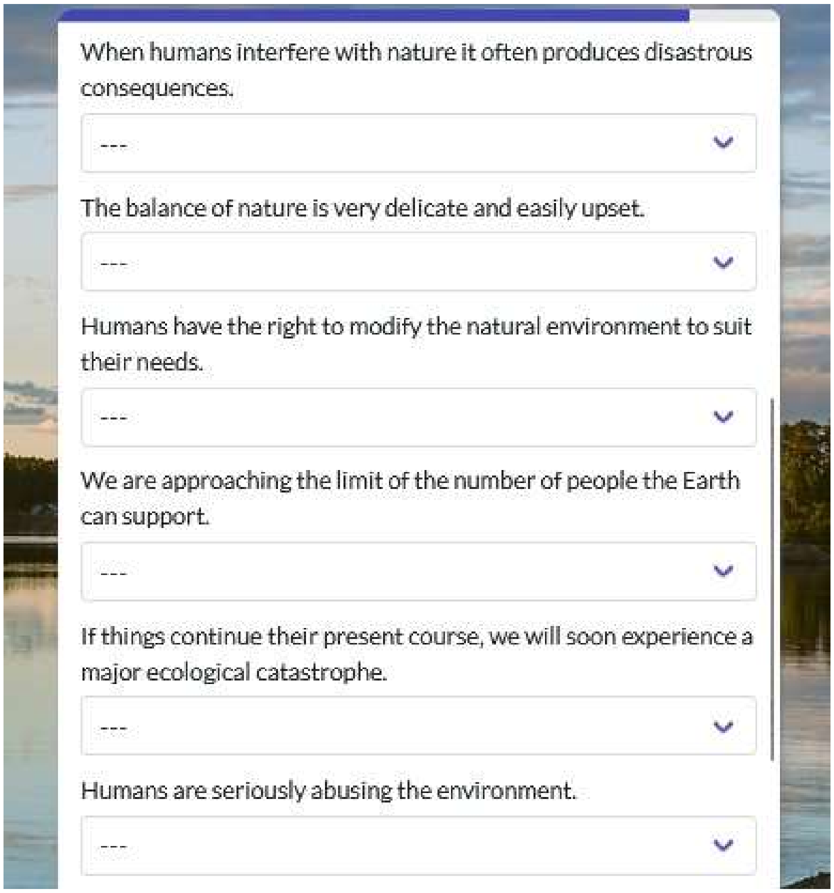

Figure S12. The continuation of the sixth section.

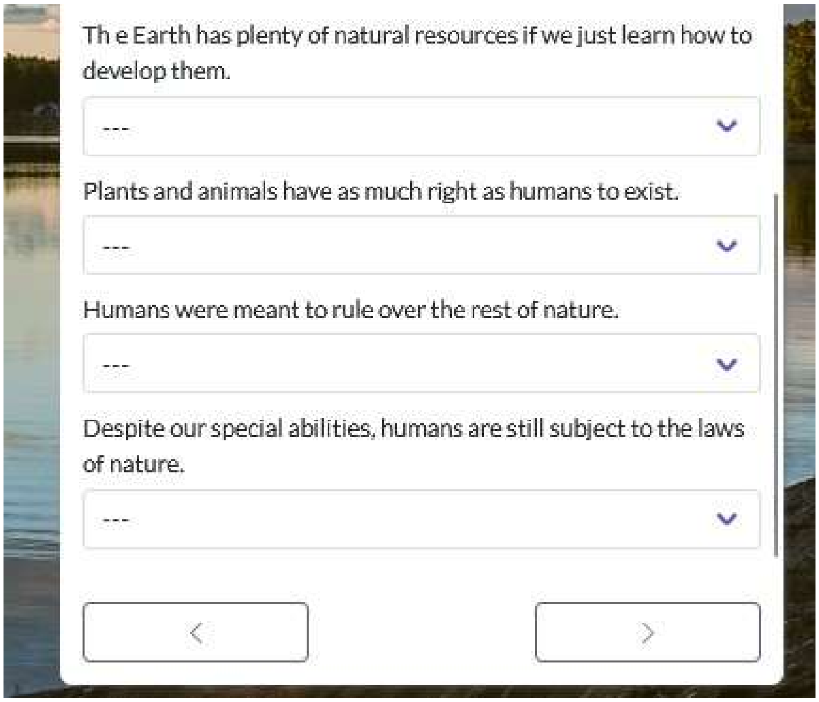

Figure S13. The rest of the sixth section.

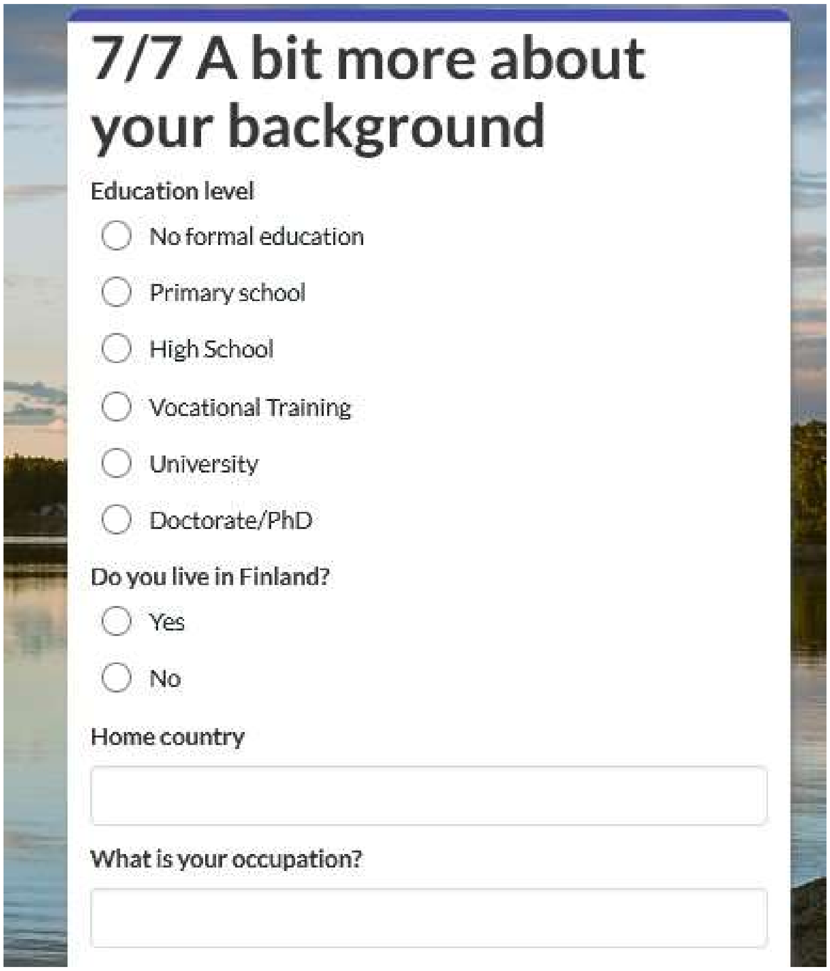

Figure S14. In the seventh section, additional questions were asked about the respondent's background information, such as education, place of residence and occupation.

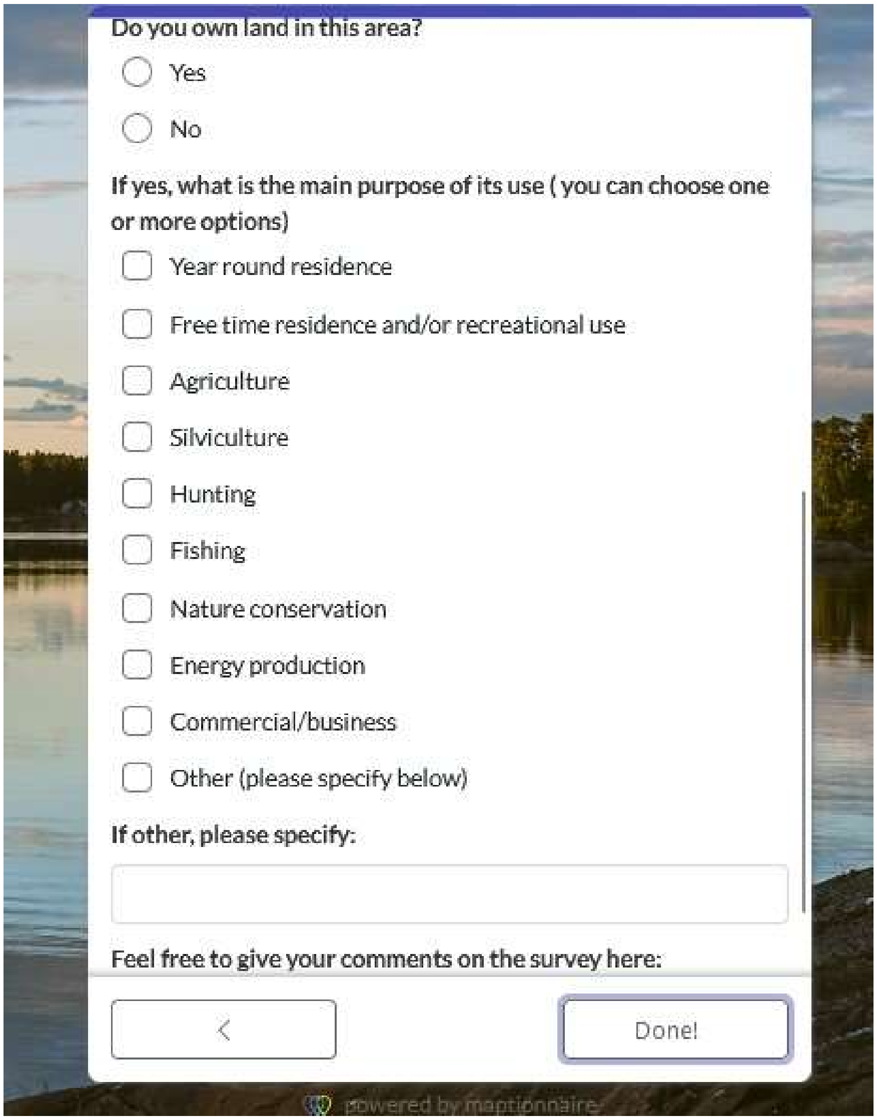

Figure S15. The rest of the seventh section and end of the survey.

Table S1. Description and examples of open responses describing landscape values for the seven different categories identified through content analysis.

Category	Description	Examples
Beautiful landscape or landmark	Respondents appreciate archipelago landscapes, especially their diversity, the character of the archipelago, beauty, or coastal landscapes.	"Beauty. New landscapes every day" (tourist female, 27)<br><br>"The sea and beautiful landscapes and their preservation as habitats for animals." (recreationist male, 36)
Wilderness and pristine	Respondents value the wilderness and naturalness of the archipelago, especially its untouched, free, and undeveloped environment.	"Untouched landscapes, idyllic cottages, and pristine archipelago." (recreationist male, 42)<br><br>"The most important aspect of the landscape is the sea and the islands, whose shores are not built full of cottages. Especially the outer archipelago is enchanting because it is more in a natural state, and there are many uninhabited islets, etc.(...)." recreationist female, 40)
Biodiversity	Respondents value the biodiversity of nature, especially birds and overall biological diversity.	"Rare plants and their conservation are a top priority. The preservation of birdlife and fish stocks (...)"(recreationist female, 34)<br><br>"Archipelago, sea, and the characteristic vegetation of the archipelago sea." (recreationist male, 57)<br><br>"The sea and coastal cliffs, gazing into the distance is relaxing, seeing different wildlife than in urban areas (...)." (tourist male 36)
Cultural history or heritage	Respondents value the cultural heritage and habitat of the archipelago, such as traditional archipelago villages and architecture, cultural landscapes and heritage, vibrant local communities, traditional livelihoods, and a simple way of life, etc.	"Traditional archipelago settlements, naturalness." (local male, 55)<br><br>"The people, the 'islanders,' if one can say so, those who live, reside, and work in the archipelago, who take care of and support to the archipelago to give it all these typical features (...)"(recreationist female, 63)
Silence	Respondents value the tranquillity experienced in the archipelago, especially the inner tranquillity, the surrounding silence, spaciousness, and the limited number of people.	"Seascapes and the tranquillity of the archipelago." (local female, 39)<br><br>"Silence. And it is far from the overly complex, urbanized consumer society where people do not appreciate each other, their environment, or themselves." (local female, 32)

Accessibility	Respondents value accessibility and opportunities for movement, such as boating routes, freedom of movement, and cycling routes.	“Diversity, the possibility of moving on water (...)” (recreationist female, 53)<br><br>“The freedom to move by boat wherever one wants” (recreationist male, 47)
Outdoor recreational activities	Respondents value the potential outdoor activities in the archipelago.	“Outdoor Activities, opportunities, and cycling routes." (recreationist male, 38)<br><br>“Uninhabited islands and rocks. The entire area, sea, tranquillity, recreational opportunities." (recreationist female, 50)

Table S2. Description and examples of open responses describing tourism development preferences for the eight different categories identified through content analysis.

**Tourism development preference**	**Quote**
Desired small scale tourism development	“The nature of the Archipelago Sea is delicate and large numbers of tourists spoil it. I hope that tourism remains small scale so that nature preserves its uniqueness(..) (recreationist female, 54)
Negative environmental impacts of tourism	“The large masses of people and thus the use of nature and emissions on the environment are worrying. I also fear for the peace of nature if tourism in the archipelago increases” (recreationist female, 33)<br><br>“Worry about the growing boat traffic and the noise it causes, which takes the peace away from living things and also from people (…)” (recreationist female, 63)
Increasing tourism	"The Archipelago Ring Road has increased traffic and the number of people in the area tremendously over the past 15 years. I understand that it brings income to locals, but can nature withstand it? In my opinion, the value of the area lies precisely in having few people. Will the value be preserved as the number of tourists grows?" (recreationist female, 45)
Nature and local tourism planning	“The balance between the diversity of the vulnerable archipelago nature and human beneficial and recreational use. How to protect nature values as tourism increases and develops? Perhaps nature tourism can play a part in this” (local female, 22)
Challenges of mobility and accessibility	“Those who live all year-round in the archipelago should always have priority on the ferries over the tourists. We are a family with children and if we drive our children on a hobby to the mainland,

	we do not know if we can get home on the next ferry even though we live all year round at Rosala. This is completely absurd that all tourists/summer guests should be equated with residents.” (local male, 38)
Growing and positive economic impacts	"Tourism should be allowed to increase so that it would be profitable for tourism entrepreneurs." (local male, 45)
Lack of services and products	"(...) Unfortunately, it's impossible to serve authentic archipelago food because seals and seabirds cannot be on the menus, and it's cheaper for entrepreneurs to buy fish from Estonia or farmed salmon from Norway (...)" (local female, 35)<br><br>"It would be nice to have more rental cottages. It would also be nice to have more camping areas." (recreationist female, 60)
Supporting local communities	“In order to promote the profitability of the archipelago for residents, large scale investments should be made to strive to extend the tourist season and the tourism season alike. As it stands now, the government's plans for the archipelago are only to make it a resort, rather than a residential area.” (local male, 39)

Table S3. Classification and justification of mapped landscape values according to the intrinsic, relational and instrumental values presented at the IPBES Value Typology (2022).

Landscape values (map markings)	IPBES value category	Justification
Beautiful landscape or landmark	Intrinsic	The aesthetic and uniqueness value of the Archipelago Sea is understood as end-in-themselves, not substitutable and independent of humans as valuers (IPBES, 2022).
Outdoor activities	Relational/Instrumental	In the context of Archipelago Sea BR nature is important as it provides the possibility of outdoor activities, hence they serve as means to the end of outdoor recreation. Additionally, in the Archipelago Sea BR go beyond instrumental, they reflect the mutual relation with nature and between people through nature (IPBES, 2019, 2022) .
Cultural history or heritage	Relational	In the Archipelago Sea BR, cultural history and heritage are understood as values that result through shared relations

		with nature and through nature (IPBES, 2019, 2022). Cultural history and heritage in the Archipelago Sea BR reflect centuries of human and nature interaction, the co-evolution of landscape and human societies and values associated to individual and collective cultural identity.
Wilderness and pristine	Intrinsic	In the Archipelago Sea BR, wilderness and pristine is understood as end-in-themselves, and existing independently from humans as valuers (IPBES, 2019, 2022).
Biodiversity	Intrinsic	The biodiversity of the Archipelago Sea BR is understood not substitutable, and existing independently from individuals as valuers. (IPBES, 2019, 2022).
Social relations	Relational	In the Archipelago Sea BR, social relations are understood as values that result through mutual human connections - transcending utilitarian purposes - both with nature and among individuals, facilitated by experiences in nature (IPBES, 2019, 2022).
Silence	Relational/Instrumental	In the Archipelago silence is valued as instrumental, as means to satisfy human preferences, such as calmness. Thus, something that is important to satisfyy some human end or preferences (Pascual et al., 2017). In addition, silence is also understood in the Finnish and Archipelago Sea context as relational, as people and nature through silence (IPBES, 2019, 2022).
Recreational fishing, hunting and harvest	Relational/Instrumental	Fishing, hunting, harvest provide (potential) utility to humans, to satisfy human needs IPBES 2019, 2022). Moreover, these activities are understood in Archipelago Sea BR context as relational, as people interacts with nature and through nature by fishing, hunting, and harvesting.
Learning	Relational	In the context of the Archipelago Sea learning refers to learning about nature, thence interacting with nature and with others through nature (IPBES, 2019, 2022).

Table S4. Classification and justification of open responses describing landscape values according to the intrinsic, relational and instrumental values presented at the IPBES Value Typology (2022).

**Landscape values (open responses)**	**IPBES value category**	**Justification**
Beautiful landscape or landmark	Intrinsic	The aesthetic and uniqueness value of the Archipelago Sea is understood as end-in-themselves, not substitutable and independent of humans as valuers (IPBES, 2022).
Wilderness and pristine	Intrinsic	In the Archipelago Sea BR, wilderness and pristine is understood as end-in-themselves, not substitutable and independent of humans as valuers (IPBES, 2019, 2022).
Biodiversity	Intrinsic	The biodiversity of the Archipelago Sea BR is understood not substitutable, and existing independently from individuals as valuers. (IPBES, 2019, 2022).
Cultural history or heritage	Relational	In the Archipelago Sea BR, cultural heritage and livelihoods are understood as values that result through shared relations with nature and through nature (IPBES, 2019, 2022). Cultural history and heritage in the

		Archipelago Sea BR reflect centuries of human and nature interaction, the co-evolution of landscape and human societies and values associated to individual and collective cultural identity.
Silence	Relational/Instrumental	In the Archipelago silence is valued as instrumental, as means to satisfy human preferences, such as tranquillity and the absence of noise. Thus, something that is important to satisfyy some human end or preference (Pascual et al., 2017). In addition, silence is also understood in the Finnish and Archipelago Sea context as relational, as people and nature through silence (IPBES, 2019, 2022).
Accessibility	Instrumental	In the Archipelago accessibility valued as instrumental, as means to satisfy human mobility preferences. Thus, something that is important to satisfyy some human needs or preferences (Pascual et al., 2017).
Outdoor recreational activities	Relational/Instrumental	In the context of Archipelago Sea BR nature is important as it provides the possibility of outdoor activities, hence they serve as means to the end of outdoor recreation. Additionally, in the Archipelago Sea BR go beyond instrumental, they reflect the mutual relation with nature and between people through nature (IPBES, 2019, 2022) .

Table S5. Open answers tourism development preferences.

**Tourism hopes and concerns (open responses) X=22.202, p=0.075**	**All (n=422) n=829**		**Locals (n=105) n=233**		**Recreationist (n=195) n=379**		**Tourists (n=122) n=217**	
	**freq.**	**%**	**freq.**	**%**	**freq.**	**%**	**freq.**	**%**
Desired small scale tourism development	193	23.3	27	11.6	53	14.0	22	10.1
Negative environmental impacts of tourism	123	14.8	42	18.0	87	23.0	64	29.5
Increasing tourism	104	12.5	26	11.1	45	12.0	30	13.8
Nature and local tourism planning	102	12.3	32	13.7	46	12.1	26	12.0
Challenges of mobility and accessibility	101	12.2	16	6.9	42	11.1	17	7.8
Growing and positive economic impacts	98	11.8	32	13.7	44	11.6	22	10.1
Lack of services and products	75	9.0	43	18.6	49	12.9	31	14.3
Support to local communities	33	4.0	15	6.4	13	3.4	5	2.3
Total	829	100.0.0	233	100	379	100.0	217	100.0

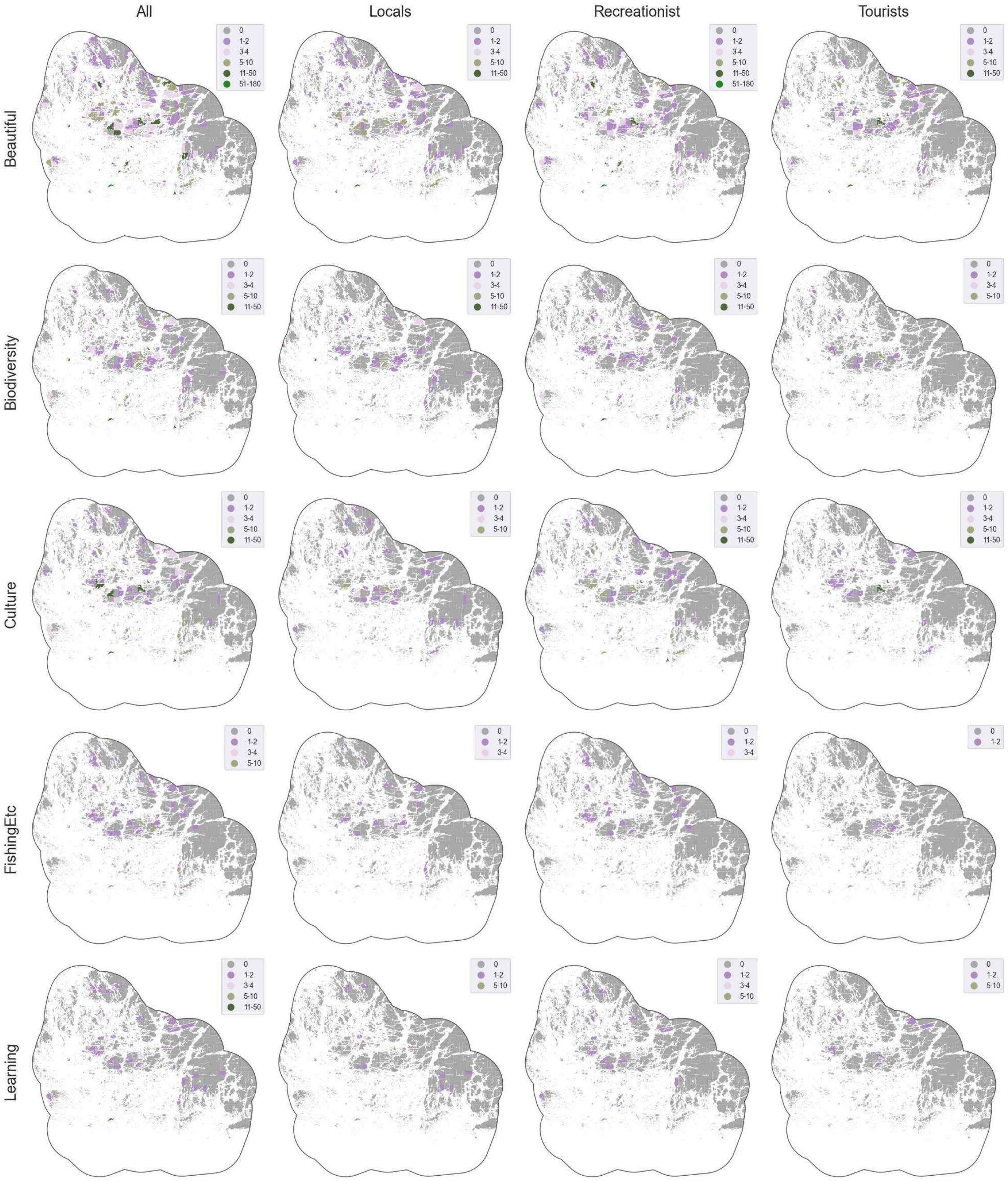

Figure S16. Landscape values (beautiful landscape or landmark, biodiversity cultural history or heritage, learning, recreational fishing, hunting and harvest) spatial distribution in the whole data and across respondent groups (n=572, 3137 locations) calculated as summed number of points per spatial unit. The study area boundaries were adjusted to align with the nearest coastline, utilizing a 2 km buffer. Data © National Land Survey of Finland and OpenStreetMap.

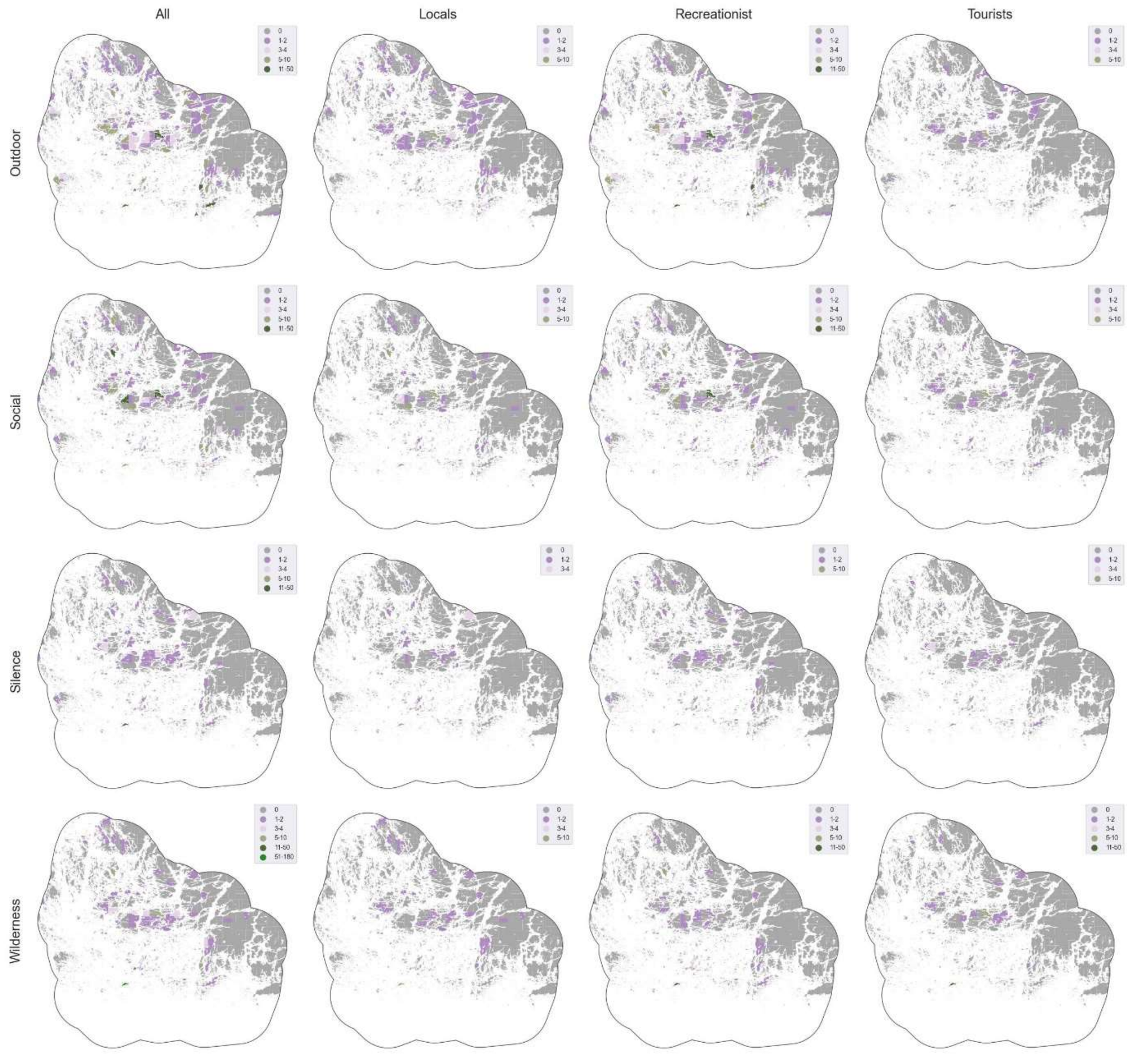

Figure S17. Landscape values (outdoor recreational activities, social relations, silence, wilderness and pristine) spatial distribution in the whole data and across respondent groups (n=572, 3137 locations) calculated as summed number of points per spatial unit. The study area boundaries were adjusted to align with the nearest coastline, utilizing a 2 km buffer. Data © National Land Survey of Finland and OpenStreetMap.

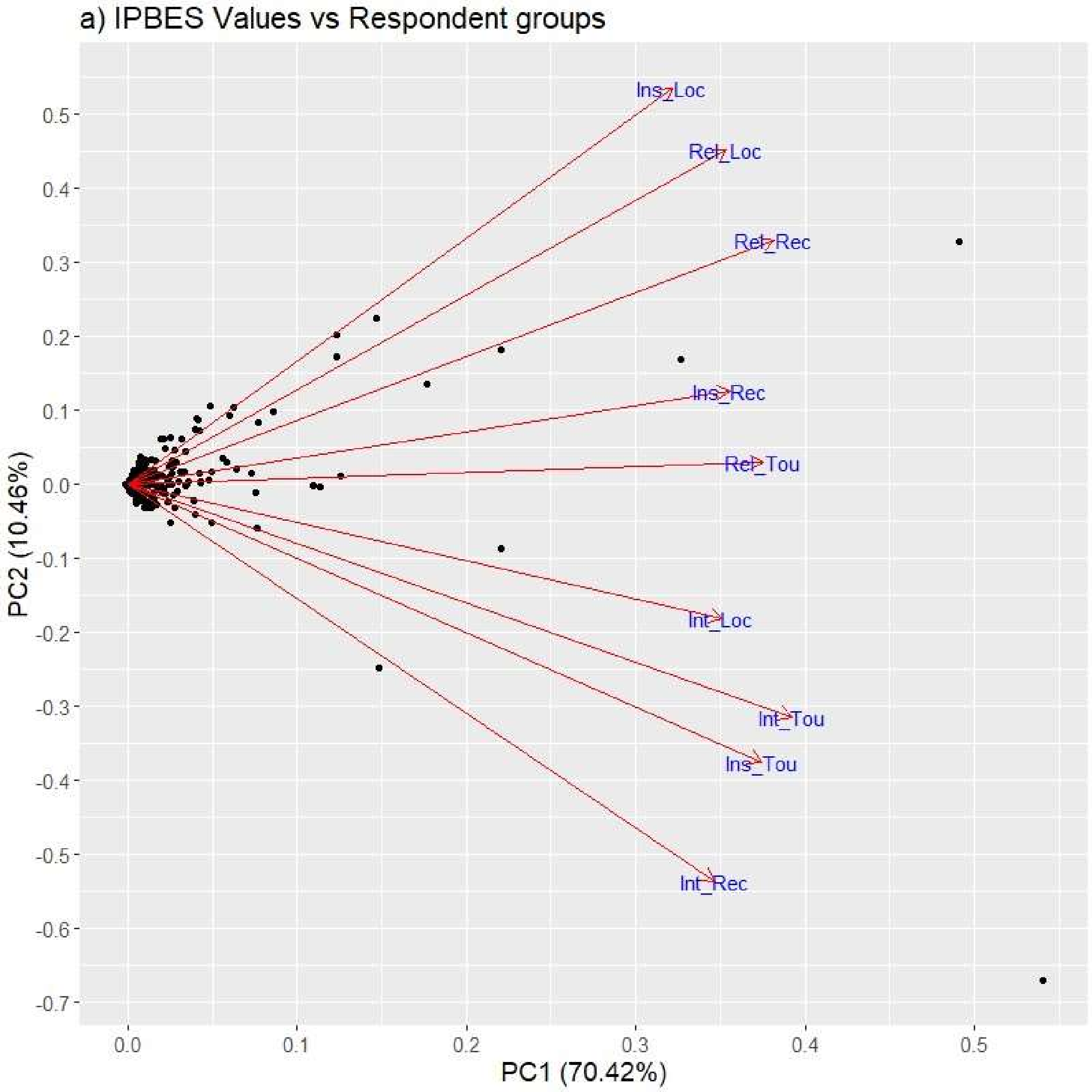

Figure S18. PCA biplot illustrating the relationships among variables and observations between the IPBES specific values and respondent groups. Each point represents an observation and the arrows indicate the direction and magnitude of each variable's contribution to the principal components.

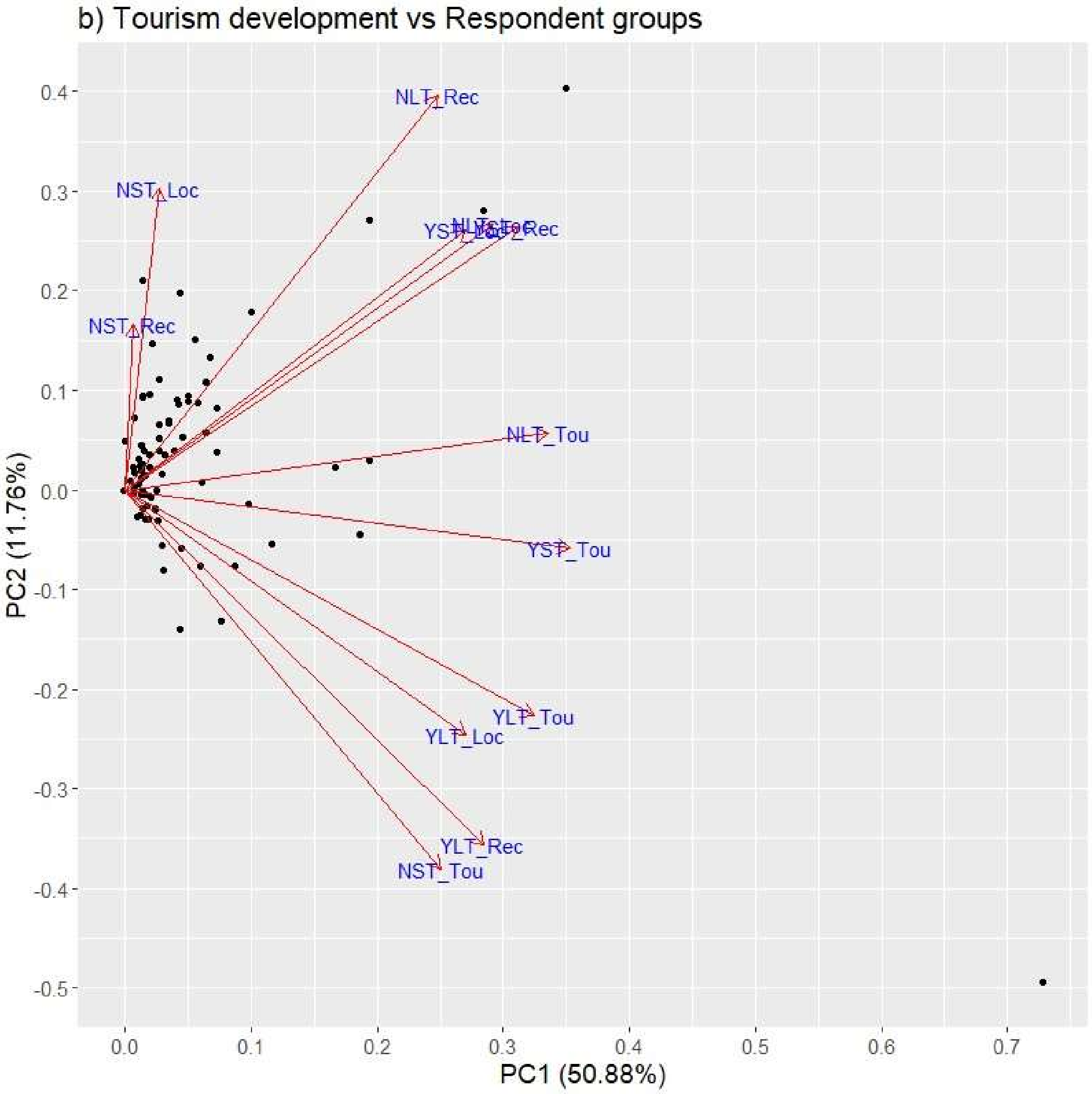

Figure S19. PCA biplot illustrating the relationships among variables and observations between tourism development types and respondent groups. Each point represents an observation and the arrows indicate the direction and magnitude of each variable's contribution to the principal components.

IPBES. (2019). *Global assessment report on biodiversity and ecosystem services of the Intergovernmental Science-Policy Platform on Biodiversity and Ecosystem Services*. https://doi.org/10.5281/ZENODO.6417333

IPBES. (2022). *Methodological assessment of the diverse values and valuation of nature of the Intergovernmental Science-Policy Platform on Biodiversity and Ecosystem Services*. https://doi.org/10.5281/ZENODO.6522523

Pascual, U., P. Balvanera, S. Díaz, G. Rgy Pataki, E. Roth, M. Stenseke, R. T. Watson, A. Mead, P. O 'farrell, R. Pandit, W. Pengue, R. N. Pichis-Madruga, F. Popa, S. Preston, D. Pacheco-Balanza, H. Saarikoski, and B. B. Strassburg. 2017. Valuing nature contributions to people: the IPBES approach. *Current Opinion in Environmental Sustainability* 26–27:7–16.

Silverman, B. W. 2018. *Density estimation: For statistics and data analysis*. Page *Density Estimation: For Statistics and Data Analysis*. CRC Press.
